# A Self‐assembled Fluid Honeycomb of Nonsymmetric Hexagons Formed by Nine Molecules in the Circumference

**DOI:** 10.1002/smll.202505094

**Published:** 2025-08-23

**Authors:** Christian Anders, Silvio Poppe, Yu Cao, Feng Liu, Carsten Tschierske

**Affiliations:** ^1^ Institute of Chemistry Martin Luther University Halle‐Wittenberg Kurt‐Mothes‐Straße 2 06120 Halle Germany; ^2^ Shannxi International Research Center for Soft Matter State Key Laboratory for Mechanical Behavior of Materials Xi'an Jiaotong University Xi'an 710049 P. R. China

**Keywords:** bolapolyphiles, liquid crystals, nonsymmetric hexagons, polygons, self‐assembly, terphenyl, tiling patterns

## Abstract

Periodic tessellations of the 2D plane by nonsymmetric polygons are of particular interest for both the 2D Kelvin problem, which is critical to system linearity and elasticity, and reticular chemistry to diverse the topological varieties. Here, a new liquid crystalline honeycomb phase, representing a monohedral tiling by nonsymmetric hexagonal cells resulting from the soft self‐assembly of nine consecutive end‐to‐end hydrogen‐bonded *p*‐terphenyl rods is reported. The new phase structure is characterized by POM, DSC as well as SAXS, with which the electron density map is reconstructed and discussed. The hexagonal cells are chiral in 2D space and give rise to a nano‐scale honeycomb resembling a *p*2*gg* tiling pattern. The findings not only pave the way to novel geometric design of reticular self‐assembled systems, but also provide nonsymmetric supramolecular motifs for 2D chiral science.

## Graphical abstract

A liquid crystalline honeycomb, representing a monohedral tiling by nonsymmetric hexagonal cells results from the self‐assembly of nine consecutive end‐to‐end H‐bonded *p*‐terphenyl derived molecules. This hexagonal cell is chiral in 2D space and gives rise to a *p*2*gg* tiling pattern, which generates a novel type of 2D tiling in ordered fluids.

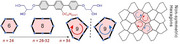

## 1 Introduction

**Figure 1:**
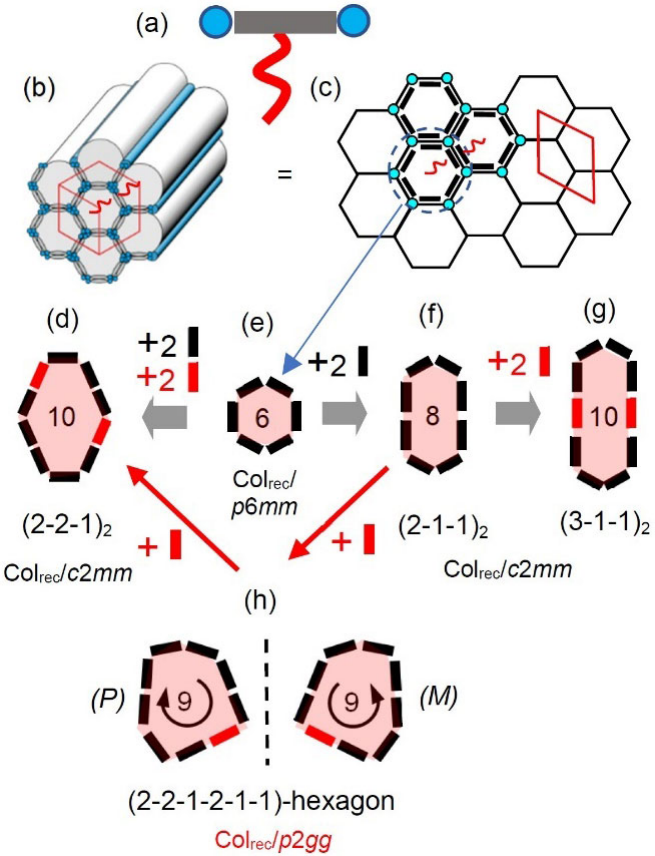
a) Schematic sketch of a T‐shaped bolapolyphile composed of a rod‐like unit with two polar end‐groups and a *n*‐alkyl side‐chain and b) hexagonal honeycomb formed by such molecules with c) cross section through the honeycomb showing the tiling pattern; d–h) show single tiles of (e) the regular hexagonal and (d, f, g) the stretched hexagonal cells with the LC phase types and the corresponding crystallographic 2D lattices;^[^ ^7^, ^12^, ^13^ ^]^ (h) shows the nonsymmetric convex hexagon formed by 9 molecules in the circumference existing in two enantiomeric forms in 2D space.

Research on 2D tiling is an attractive field as the modes of tiling are crucial to 2D material property as well as the functional design in various fields, including soft matter and reticular chemistry.^[^ ^1^ ^]^ Up to now, the majority of reported 2D tilings are based on regular polygons or a combination of regular polygons, while tessellations by nonsymmetric tiles are rare and hard to be constructed with molecular building blocks. Regular hexagons allow the periodic tiling with the largest tile area at minimal circumference.^[^ ^2^ ^]^ Therefore, such hexagonal tilings are often observed in self‐assembled systems, like patterns on solid surfaces,^[^ ^3^, ^4^ ^]^ in metal‐organic or covalent‐organic frameworks (MOFs, COFs),^[^ ^5^ ^]^ and in the liquid crystalline (LC) honeycombs formed by T‐/X‐shaped polyphilic molecules (**Figure** **1**a).^[^ ^6^, ^7^, ^8^, ^9^ ^]^ In the latter cases, rod‐like molecules with two hydrogen‐bonding glycerol end groups self‐assemble into well‐ordered fluids. The hydrogen bonds connect the rods end‐to‐end and side‐by‐side, thus forming ribbons of parallel arranged rods which fuse into honeycombs, while additional flexible and lipophilic side‐chains fill the space inside the resulting prismatic cells (Figure 1b,c). With growing side‐chain volume, the shape of the prismatic cells changes from triangular via square and pentagonal to hexagonal,^[^ ^6^, ^10^ ^]^ as found experimentally and supported by simulation work.^[^ ^11^ ^]^ Upon further chain elongation the number of molecules in the circumference further increases leading to giant honeycombs with some walls formed by pairs or even triplets of end‐to‐end connected rods.^[^ ^7^, ^8^, ^12^, ^13^ ^]^

Three types of monohedral giant honeycombs with irregular hexagonal shape are known for LC honeycombs so far, having an even number of eight or ten molecules in the circumference of the stretched hexagons (Figure 1d,f,g).^[^ ^7^, ^12^, ^13^ ^]^ In all three cases, the honeycomb shape is controlled by the side‐chain volume and its position while retaining the hexagonal shape, albeit with reduced symmetry. Upon side‐chain volume expansion, the conventional hexagon reaches its limit of storing the side‐chains. Under such circumstance, hexagons with extended sides are formed, which are composed of end‐to‐end linked pairs (2) or triplets (3) combined with individual rod‐like units (1) to form (2‐1‐1)_2_‐hexagons with 8 and (2‐2‐1)_2_‐ or (3‐1‐1)_2_‐hexagons with 10 rods in the circumference. In these honeycombs, the symmetry axis perpendicular to the polygon plane is reduced from *C*_6_ to *C*_2_, and thus the crystallographic plane group is reduced from *p*6*mm* to *c*2*mm*. Once we push the structure deformation one step further, the remaining mirror planes involving the *C*_2_ axis could be broken, leading to a tessellation by nonsymmetric convex hexagons being chiral in 2D space (Figure 1h).

Here we report the first case of such a hexagonal honeycomb with nonsymmetric cells, having an odd number of nine rod‐like units in the circumference of each prismatic cell (Figure 1h), formed by soft self‐assembly of a T‐shaped *p*‐terphenyl derived compound **1/*n*** with a single long *n*‐alkyl side‐chain (*n* = 34) at the central benzene ring (**Table** **1**).

**Table 1:** Phase transitions on heating and cooling and lattice parameters of the LC phases of compounds **1/24‐1/34**. ^a)^

	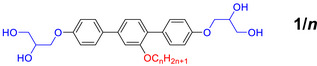	1/*n*		
1/*n*	Phase transitions *T*/°C [Δ*H*/kJmol^−1^]	*a*_hex_ /nm (*T*/°C)	*a*_rec_ *; b*_rec_ /nm (*T*/°C)	*n*_wall_
1/24	Cr 73 [31.4] Col_hex_/*p*6*mm* 182 [6.6] Iso 179 [‐6.5] Col_hex_/*p*6*mm* 40 [‐29.1] Cr	4.28 (140)	‐	1.9
1/26	Cr 67 [53.6] Col_rec_/*c*2*mm* 150 [3.3] Iso 145 [‐3.4] Col_rec_/*c*2*mm* 46 [‐36.7] Cr	‐	11.98; 4.70 (110)	2.4
1/30	Cr 71 [36.8] Col_rec_/*c*2*mm* 156 [4.5] Iso 153 [‐4.6] Col_rec_/*c*2*mm* 54 [‐40.1] Cr	‐	11.42; 4.44 (140)	2.0
1/32	Cr 77 [68.7] Col_rec_/*c*2*mm* 149 [3.5] Iso 145 [‐3.5] Col_rec_/*c*2*mm* 60 [‐52.3] Cr	‐	11.36; 4.49 (140)	2.0
1/34	Cr 87 [71.5] Col_rec_/*p*2*gg* 151 [4.3] Iso 146 [‐4.3] Col_rec_/*p*2*gg* 65 [‐60.0] Cr	‐	13.42; 9.30 (137)	2.0

^a)^ Peak temperatures in the DSC heating and cooling scans at 10 K min^−1^. Abbreviations: Cr = solid crystalline state, Iso = isotropic liquid state; Col_hex_/*p*6*mm* = hexagonal columnar LC phase (regular hexagonal honeycomb, Figure 1b,c,e); Col_rec_/*c*2*mm* = rectangular columnar LC phase with *c*2*mm* plane group representing a honeycomb composed of (2‐1‐1)_2_‐8‐hexagons (see Figure 1f); Col_rec_/*p*2*gg* = rectangular columnar phase composed of nonsymmetric (2‐2‐1‐2‐1‐1)‐9‐hexagons (see Figure 1h); *a*_hex_, *a*_rec_, *b*_rec_ = lattice parameters; *n*_wall_ = average number of molecules in the cross section of the honeycomb walls; for calculations and additional structural data, see Table S7 (Supporting Information); for the DSC traces, see Figure 3c, Figure S7 (Supporting Information) and for XRD data, see Figure 3a, Figures S12–S16 (Supporting Information) and Tables S1–S6 (Supporting Information).

## 2 Results and Discussion

### 2.1 Synthesis and Methods

**Scheme 1:**
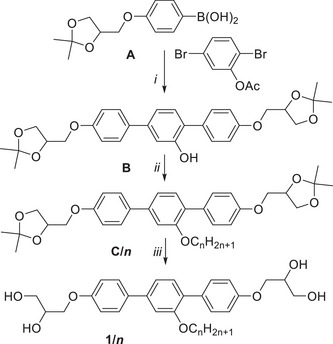
Synthesis of compounds **1/*n*** ( *n* = 24, 26, 30–34). *Regents and conditions*: *i*: 1. Pd(PPh_3_)_4_, THF, H_2_O, NaHCO_3_, reflux, 12 h; 2. NaOH, H_2_O, 25 °C, 12 h; *ii*: C_n_H_2n+1_Br, K_2_CO_3_, DMF, 80 °C, 1d; *iii*: MeOH, THF, cat. pyridinium‐p‐toluene sulfonate (PPTS), 50 °C, 12 h.

The compounds **1/*n*** were synthesized as shown in **Scheme** **1**. The *p*‐terphenyl‐2′‐ol **B** was obtained by Suzuki cross‐coupling between the glycerol‐functionalized benzene boronic acid **A** with 2,5‐dibromophenylacetate followed by basic hydrolysis of the acetyl group, as reported previously.^[^ ^11^ ^]^ Alkylation with long‐chain *n*‐alkylbromides, followed by acid hydrolysis of the acetonide groups, yielded the compounds **1/*n*** with *n* = 26 and 30–34 (*n* = 28 was skipped because **1/26** and **1/30** turned out to form identical phase structures and no additional new phase structure could be expected for this intermediate compound). All compounds were investigated by polarizing optical microscopy (POM), differential scanning calorimetry (DSC), and by small‐ and wide‐angle X‐ray scattering (SAXS and WAXS). The synthesis, purification, and investigation methods are described in detail in Sections S1,S2 (Supporting Information).^[^ ^11^, ^14^, ^15^ ^]^

### 2.2 Honeycomb Deformation Depending on Side‐Chain Length

**Figure 2:**
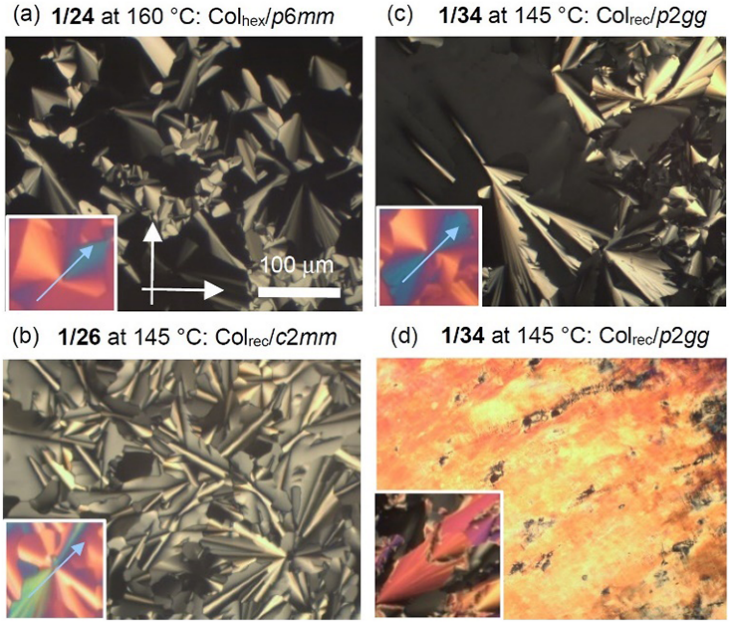
a–c) Typical optical textures as observed between two plane microscopy glass plates (≈20 µm distance) between crossed polarizers (white arrows) in the different honeycomb LC phases of compounds **1/*n*** at the indicated temperatures; the directions of the polarizers are shown in (a); the dark areas in (a) and the low birefringent areas in (b,c) show uniform alignment of the honeycomb long axis perpendicular to the substrate surfaces, they appear completely dark for the uniaxial Col_hex_ phase (and remain completely dark by rotation of the sample between the crossed polarizers), and are weakly birefringent in the optical biaxial Col_rec_ phases (here the brightness changes by rotation of the sample). Insets at the bottom show the textures with additional λ‐retarder plate, indicating negative birefringence in all LC phases by the blue shift along the major (slow) indicatrix direction, indicated by the blue arrow (for textures of **1/30** and **1/32**, see Figure S9, Supporting Information); d) shows the texture of **1/34** immediately after shearing; the inset shows the spontaneously reorganized fan‐texture after 30 min storage, both confirming the fluidity of the LC honeycomb (see also Figures S9,S10,S11, Supporting Information).

**Figure 3:**
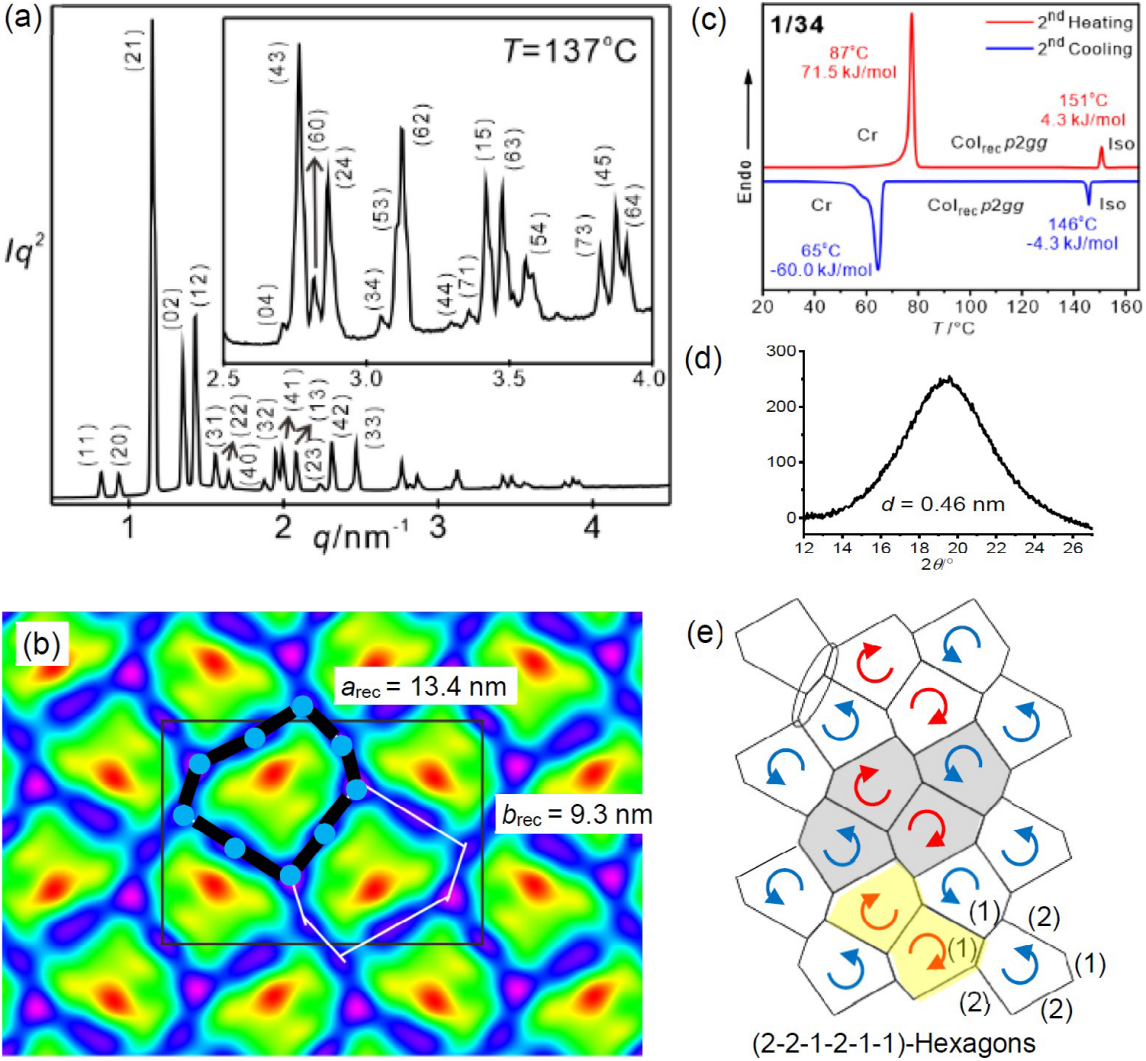
Compound **1/34**, a) SAXS diffractogram of the Col_rec_/*p*2*gg* phase at 137 °C (see also Tables S5, S6, Supporting Information); b) the ED map reconstructed from this pattern (phase choice π0π0π0π00ππ000ππ000π) with overlaid molecules around one cell (black rods and blue circles for the bolaamphiphilic cores, the side chains are not shown), c) DSC heating and cooling traces at 10 K min^−1^; d) diffuse WAXS at 120 °C and e) periodic tiling by 9‐hexagons; the grey area indicates the translational unit and yellow indicates a *C*_2_‐symmetric antiparallel pair; the arrows show the direction of the sequence (2‐2‐1‐2‐1‐1) red: *(P)* and blue: *(M)*.

Three distinct enantiotropic (thermodynamically stable) LC phases were observed over broad temperature ranges (64–87 K) (see Table 1). Between crossed polarizers, birefringent optical textures can be observed for all compounds, confirming the presence of long‐range order. All compounds exhibit spherulite‐like and fan‐like textures, as typical for LC phases with 2D lattice (see **Figure** **2**a–c, Figure S9, Supporting Information).^[^ ^16^ ^]^ In all cases, the birefringence is negative, in line with honeycomb structures, where the major π‐conjugation pathway (the slow axis along the *p*‐terphenyl core) is perpendicular to the column direction, as confirmed by investigation with a λ‐retarder plate (insets in Figure 2a–c).^[^ ^14^ ^]^ The fluidity of the LC state leads to an easy distortion of the textures, flowing under slight mechanical stress (Figure 2c→d, Figure S11, Supporting Information). For all of them, the wide‐angle X‐ray scattering (WAXS) is completely diffuse with a maximum at *d* ≈ 0.45 – 0.46 nm (see Figures 3d and S12–S15, Supporting Information), confirming LC phases where the individual molecules do not have fixed positions.

The plane group of the 2D lattice and the lattice parameters were determined by small‐angle X‐ray scattering (SAXS). Compound **1/24** with the shortest chain forms a hexagonal columnar LC phase with *p*6*mm* plane group and a lattice parameter *a*_hex_ = 4.28 nm, almost the same as reported for the shorter homologue **1/22** ^[^ ^11^ ^]^ (*a*_hex_ = 4.24–4.32 nm), and typical for a regular hexagonal honeycomb (Figure S12, Supporting Information). The effective length of the *p*‐terphenyl based core‐unit between the ends of the glycerol units (*L*_mol_) can be estimated according to *L*_mol_ = *a*_hex_/√3 ≈ 2.5 nm, which is in good agreement with the measured values using space‐filling molecular models (*L*_min_ = 2.3 nm; *L*_max_ = 2.6 nm, see Figure S17, Supporting Information).^[^ ^14^ ^]^ The hexagonal lattice is additionally supported by the uniaxiality of this columnar LC phase, as confirmed by POM (Figure 2a).

The subsequent even numbered homologues with longer side‐chains, compounds **1/26**, **1/30,** and **1/32** form another – in this case biaxial – LC phase (Figure 2b, Figure S9, Supporting Information) with 2D lattice. The SAXS patterns of these compounds can be indexed to a centered rectangular lattice with *c*2*mm* plane group (Figures S13–S15, Tables S2–S4, Supporting Information). For compound **1/26**, as example, the parameter *a*_rec_ = 11.98 nm corresponds to ≈5 times *L*_mol_; while the parameter *b*_rec_ = 4.70 nm is close to √3*L*_mol_, indicating a (2‐1‐1)_2_‐hexagonal honeycomb with 8 molecules forming the circumferences of the cells (Figure 1f).^[^ ^13^ ^]^ Expanding side chain volume reduces both lattice parameters a bit to *a*_rec_ = 11.36‐11.42 nm and *b*_rec_ = 4.44–4.49 nm for **1/30** and **1/32**. From these parameters and the molecular volumes, it can be estimated that about two back‐to‐back organized molecules are organized on average in the lateral cross‐section of each of the honeycomb walls (*n*_wall_ ≈ 2, see Table 1 and Table S7, Supporting Information). While for compounds **1/30** and **1/32** *n*_wall_ = 2.0 as expected, the larger number *n*_wall_ = 2.4 for **1/26** is attributed to its proximity to the transition to the Col_hex_/*p*6*mm* phase with smaller regular 6‐hexagonal cells. To completely fill the larger 8‐hexagons some more alkyl chains are required which are provided by additional molecules in the lateral diameter of the honeycomb walls (leading to the increased lattice parameters of **1/26** compared to **1/30** and **1/32**), while for the smaller 6‐hexagons of **1/24** which are overcrowded it is a bit smaller than two (*n*_wall_ = 1.9) and this number becomes *n*_wall_ = 2.0 again for compound **1/22** with a shorter side‐chain.^[^ ^11^ ^]^

### 2.3 Irregular Hexagonal Honeycomb of the *p*2*gg* Phase of **1/34**

Compound **1/34** with the longest chain forms a rectangular columnar phase over a wide temperature range (**Figure** **3**c), too, but with *p*2*gg* lattice and even larger lattice parameters of *a*_rec_ = 13.42 and *b*_rec_ = 9.30 nm (Table 1). The electron density (ED) map, reconstructed from the synchrotron‐SAXS pattern in Figure 3a and shown in Figure 3b indicates a tessellation by nonsymmetric hexagonal tiles having three walls formed by end‐to‐end connected molecular pairs and three walls formed by single molecules in the sequence (2‐2‐1‐2‐1‐1). The low ED aliphatic interior of the prismatic cells (red, yellow, green) is separated by high ED walls (blue) containing the *p*‐terphenyl cores and the glycerols (purple) at the three‐way junctions and the two‐way junctions in the middle of the pairs of molecules in the three longer walls. The side length is ≈5 nm for the long and ≈2.2 nm for the short sides, in good agreement with the double or the single molecular length, respectively. There are ≈37 molecules per unit cell, i.e., two back‐to‐back aligned *p*‐terphenyl rods form the 18 honeycomb walls in each unit cell (*n*_wall_ = 2.0, Table 1, Table S7, Supporting Information), which fits nicely to the other compounds. There is no substantial increase of *n*_wall_ at the *c*2*mm*‐*p*2*gg* transition from 8‐ to 9‐hexagons, because the difference in cell size is smaller than from 6‐ to 8‐hexagons at the *p*6*mm*‐*c*2*mm* transition (for the effect of temperature on *n*_wall_, see Figure S16, Supporting Information). Overall, the formation of this new polygon tiling with an odd number of nine molecules in the circumference is mainly driven by the increased volume of the C_34_ side chain compared to **1/32**.

### 2.4 Effects of Side‐Chain Position: 9‐Hexagon versus10‐Hexagon Competition

**Figure 4:**
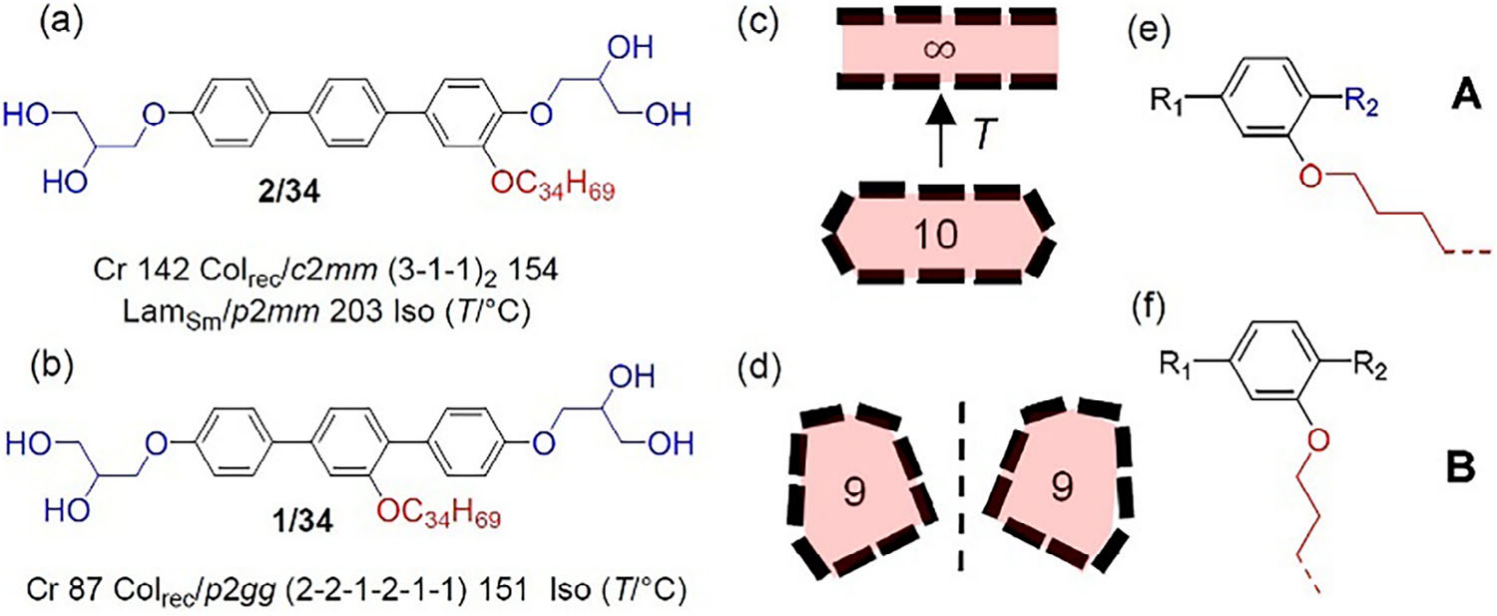
a,b) Effect of the position of the *n*‐alkyl chain on the LC self‐assembly of compounds **1/34** and **2/34**,^[^ ^13^ ^]^ c,d) sketches of the LC phase structures and e,f) conformational isomers **A** and **B** showing the different preferred directions of the lateral *n*‐alkyl chains at the *p*‐terphenyl core.^[^ ^17^ ^]^

In **Figure** **4** compound **1/34** is compared with the previously reported isomeric compound **2/34,** having the same C_34_ chain, but in this case positioned at the molecular periphery adjacent to one of the glycerol units.^[^ ^13^ ^]^ Shifting the alkyl chain from the periphery to the middle of the molecules reduces all transition temperatures by ≈50 K, which is especially relevant for the melting point, allowing the occurrence of the LC phases much closer to ambient temperature in the case of **1/34** (Figure 4a,b). This is attributed to the larger distortion of parallel rod‐packing by lateral substituents in the middle of the *p*‐terphenyls. Moreover, the 9‐hexagon tiling of **1/34** is for the isomeric compound **2/34** replaced by the more symmetric (3‐1‐1)_2_‐type of stretched 10‐hexagon tiling (Col_rec_/*c*2*mm*), accompanied by a lamellar phase (Lam_Sm_/*p*2*mm*), where the honeycombs are unfolded and the *p*‐terphenyls form quasi infinite (∞) layers with the *p*‐terphenyls being aligned parallel to each other and parallel to the layer planes (see Figure 4a,c).^[^ ^13^ ^]^ This lamellar phase is completely absent in the series **1/*n***.

Due to the different side‐chain positions, there are different energies for conformations **A** and **B** (Figure 4e,f).^[^ ^17^ ^]^ For series **2/34** with the OC_34_H_69_ substituent adjacent to the glycerol (R_1_ = Ph, R_2_ = OCH_2_‐…) there is a preference for conformation **A**, supporting the alignment of the *n*‐alkyl chains parallel to the *p*‐terphenyl rods, thus giving rise to cell stretching with formation of the elongated and still symmetric (3‐1‐1)_2_‐10‐hexagons (Col_rec_/*c*2*mm*) together with a lamellar phase (Lam_Sm_/*p*2*mm*). For **1/34** the chain positioned in the middle of the *p*‐terphenyl experiences a significant steric repulsion with the adjacent benzene ring (R_1_ = R_2_ = Ph), which provides a larger contribution of the T‐shaped conformer **B**, leading to a stronger inclination between chains and *p*‐terphenyls, which requires a more isotropic way of cell expansion. This removes the Lam phase completely and leads to a reduced aspect ratio of the polygons. Because monohedral tilings by polygons larger than hexagons are impossible,^[^ ^2^, ^18^ ^]^ the hexagon has to be retained, but it becomes nonsymmetric by expansion of one of the (1)‐sides of the 8‐hexagon to a (2)‐side in the 9‐hexagon (Figure 1f→h).

**Figure 5:**
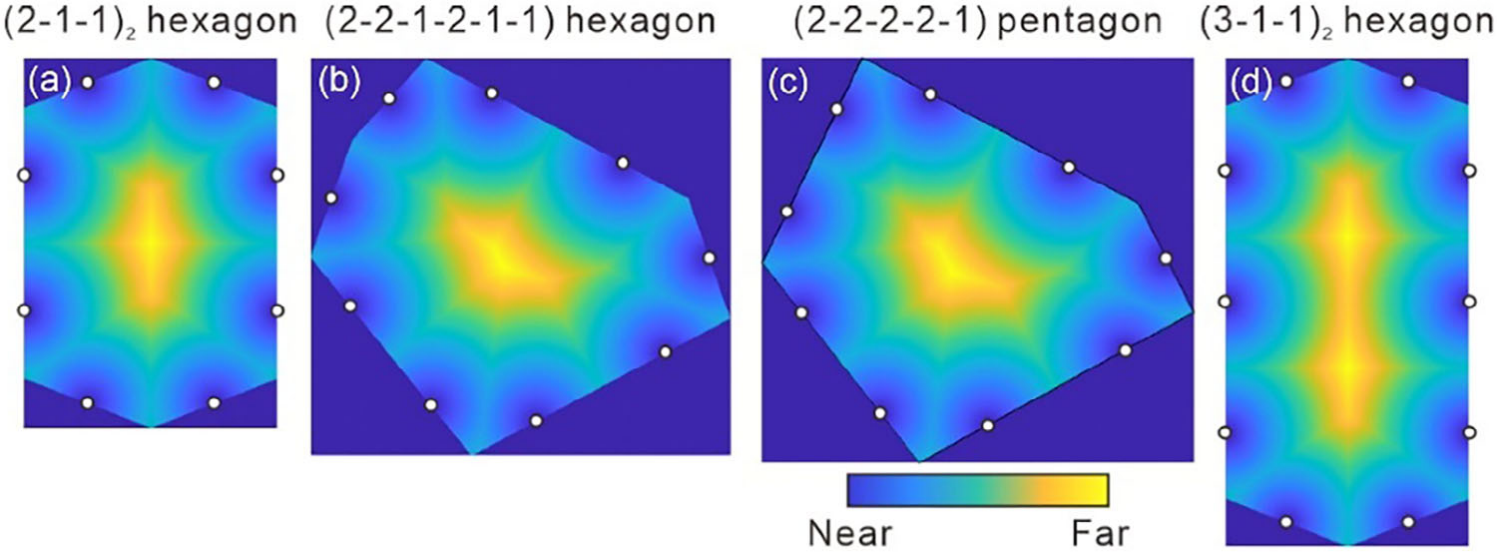
The distance distribution maps of the four different tilings under discussion, namely a) the (2‐1‐1)_2_‐8‐hexagon (*c*2*mm*), b) the (2‐2‐1‐2‐1‐1)‐9‐hexagon (*p*2*gg*), c) the hypothetical (2‐2‐2‐2‐1)‐9‐pentagon and d) the (3‐1‐1)_2_‐10‐hexagon (*c*2*mm*). The white circles indicate centers of molecules along the edges. The light‐yellow region is furthest away from all molecules along the edges; note that all maps refer to compounds **1/*n*** with alkyl chains close to the middle of the *p*‐terphenyls.

Restrictions concerning possible combinations of side‐lengths and vertex‐angles allow only three types of non‐symmetric hexagonal shapes being capable of tiling the plane without overlaps or gaps.^[^ ^18^ ^]^ That one being most compatible with a tiling by 9 (almost) identical units is the *type‐2* hexagon,^[^ ^18a,b^ ^]^ shown in Figures 1h,3b,e (see also the explanations in the caption to Figure S18d,e, Supporting Information). However, even this tiling requires some deviations of the individual side lengths from being exactly one or two *p*‐terphenyls (see Figure S18c, Supporting Information). Such deviations are allowed within certain limits (≈2.0–2.6 nm) by the flexibility of the dynamic and cooperative hydrogen bonding between the glycerols at the junctions.^[^ ^19^ ^]^ The combination of primary and secondary OH groups, and ether oxygens allows different H‐bonding motifs, and the three consecutive single bonds between the hydrogen bonding sites allow different conformations, which all together enable different glycerol group conformations (Figure S17, Supporting Information) and packing modes for the adjustment of the junction shape and geometry. The problem of adjusting the polygon side length is reduced in the more symmetric stretched (3‐1‐1)_2_‐type of 10‐hexagons where the vertex angles are less strictly fixed, and in addition, chains and rods can more easily align parallel. However, in this case, an energetic penalty results from the reduced space filing in the larger 10‐hexagons (see **Figure** **5**d). This can be compensated by a slight variation of the vertex angles and by increasing the number of molecules organized in the diameter of the honeycomb walls (*n*_wall_ = 2.2 for **2/34** ^[^ ^13^ ^]^), thus providing more chains to fill the space in these larger cells. This lateral wall expansion is easier for peripheral substituted cores (**2/34**), where the chains provide less steric hindrance for the parallel packing of more than exactly two *p*‐terphenyls side‐by‐side. Thus, conformational differences and restrictions in core‐packing support 9‐hexagons for **1/34** and 10‐hexagons for the isomeric compound **2/34**.

There are additional volume effects of hydrogen bonding and side‐chain volume distribution. Though the hydrogen bonding networks between the glycerols are highly dynamic and do not provide specific junction geometries, the cooperativity of this hydrogen bonding prefers larger over smaller hydrogen bonding networks,^[^ ^19^, ^20^ ^]^ meaning that 2‐way junctions (connecting the terphenyls in the (2)‐walls) are enthalpically disfavored with respect to the 3‐way junctions and thus tend to be minimized. This provides an additional advantage of the 9‐hexagon with only three 2‐way junctions over the (3‐1‐1)_2_‐10‐hexagon, having four of them at the same number of six 3‐way junctions. However, for the (3‐1‐1)_2_‐10–hexagon of **2/34,** this effect is reduced due to the increased *n*_wall_ = 2.2^[^ ^13^ ^]^ which expands the junctions a bit, thus contributing to its stabilization in this case.

**Figure 6:**
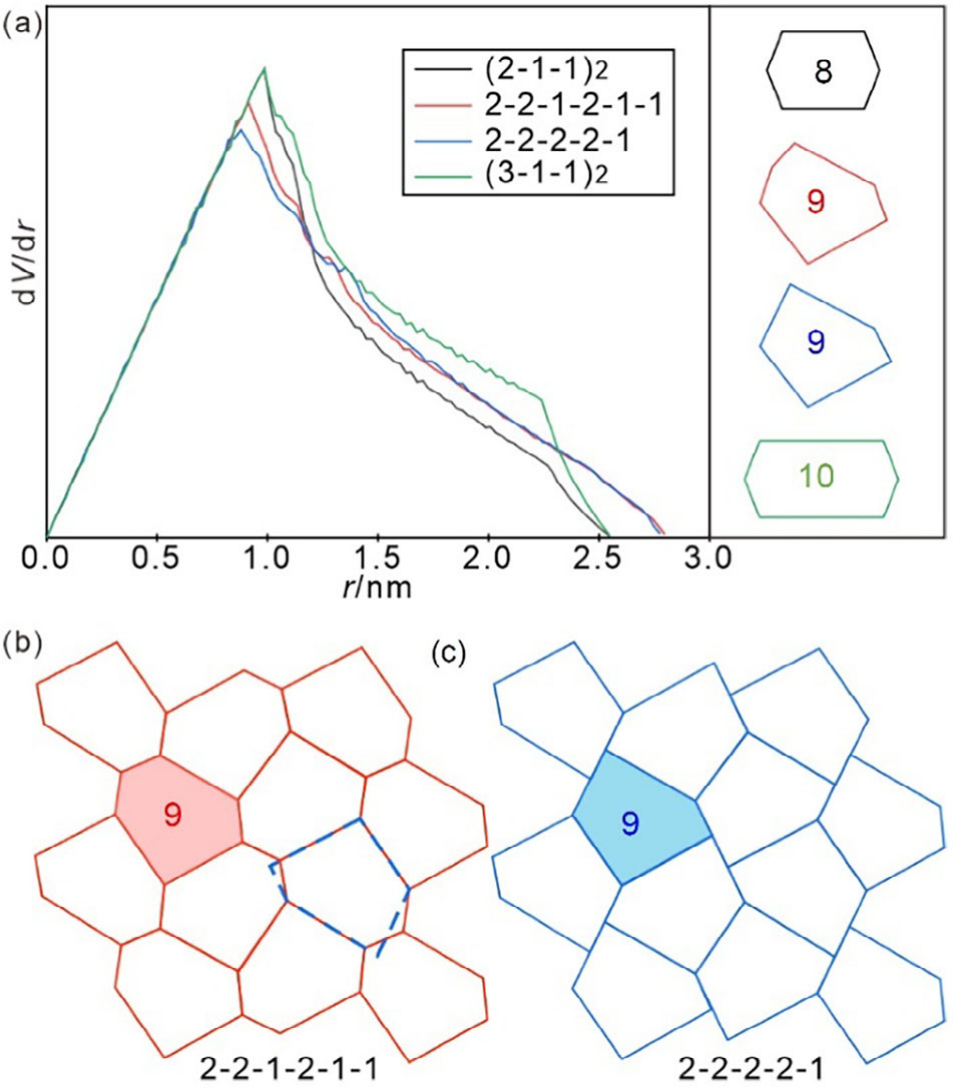
a) The molecular radial volume distribution d*V*/d*r* for the four types of tiles, (2‐1‐1)_2_‐8‐hexagon (*c*2*mm*), (2‐2‐1‐2‐1‐1)‐9‐hexagon (*p*2*gg*), (2‐2‐2‐2‐1)‐9‐pentagon, and (3‐1‐1)_2_‐10‐hexagon (*c*2*mm*). The position difference of alkyl chain between compounds **1/*n*** and **2/*n*** just has minor influence (≈0.1 nm) on the curve ends and almost no influence (<1%) on occupied area; b) shows the edge‐to‐edge (2‐2‐1‐2‐1‐1)‐9‐hexagon tiling in comparison to c) the hypothetical non‐edge‐to‐edge (2‐2‐2‐2‐1)‐9‐pentagon tiling.

**Figure** **6**a shows the molecular radial side‐chain volume distribution d*V*/d*r* for the three types of irregular hexagon tilings under discussion, together with a hypothetical (2‐2‐2‐2‐1)‐9‐pentagon tiling (blue), which will be discussed in the next section. The area occupied by the d*V*/d*r* curve represents the alkyl chain volume of the molecule. Normalized to the (2‐1‐1)_2_‐8‐hexagon, the (2‐2‐1‐2‐1‐1)‐9‐hexagon requires 106.5% alkyl chain volume, and the (3‐1‐1)_2_‐10‐hexagon requires 113.8%. If we take **1/32** as the upper limit, i.e., fully filled 8‐hexagons, the alkyl chain volume ratio between **1/32** and **1/34** is 106.3%. This volume expansion is close to the 8‐hexagon to 9‐hexagon cell expansion. Such similarity supports the exclusive formation of the 9‐hexagon by **1/34** (Figure 4), i.e., this nonsymmetric 9‐hexagon is favored by the volume effect. However, since 34 carbons is already a long alkyl chain, it also has a strong tendency of parallel packing, i.e., denser packing, which tends to favor the stretched 10‐hexagons, requiring shorter *r* to fill the tiles. As noted above, for **1/34** the packing is more arbitrary as alkyl chains are in the middle of the aromatic core, favoring the volume effect, inducing the 9‐hexagon over the denser packed 10‐hexagon of **2/34**. In short, upon alkyl chain volume expansion, the 6‐hexagon (*p*6*mm*) to (2‐1‐1)_2_‐8‐hexagon (*c*2*mm*) transition relies more on enthalpy with more ordered chain and core packing in the (2‐1‐1)_2_‐8‐hexagons. Upon further alkyl chain elongation, the competition between symmetric (3‐1‐1)_2_‐10‐hexagons and nonsymmetric (2‐2‐1‐2‐1‐1)‐9‐hexagons is significantly influenced by entropy.

### 2.5 9‐Hexagon versus 9‐Pentagon Tiling

Removing the slight bend of the edge between the two short adjacent walls with single molecular length of the 9‐hexagon tiling (Figure 6b) would give a periodic (2‐2‐2‐2‐1)‐type 9‐pentagon tiling with one vertex positioned in the middle of the thus created additional (2)‐side, leading to a non‐edge‐to‐edge tiling (Figure 6c), having the same *p*2*gg* plane group and slightly expanded lattice parameters (≈2.5%). However, the 9‐hexagon requires less diversity of side‐length deviation from the molecular length than the 9‐pentagon (see Figure 6b,c and Figure S18b,c, Supporting Information) and is therefore favored. Especially the (2)‐sides can be adjusted closer to the double of the molecular length in the 9‐hexagon.

There are only small differences in the molecular radial volume distribution d*V*/d*r* (see Figure 6a) and degree of chain stretching (see Figure 5b,c) in these two cases. As shown in Figure 5b,c, the yellow region, which stands for the furthest position alkyl chain should reach, is slightly less curved for the 9‐hexagon if compared with the hypothetical 9‐pentagon. So, in the 9‐hexagon the alkyl chains from different molecules along the wall would assume more uniform conformations as they stretch in similar ways. For the 9‐pentagon, the alkyl chains of the molecules in the left‐bottom have to stretch slightly more than the others due to the curved yellow region. This might additionally contribute to 9‐hexagon preference. Nevertheless, the pentagonal tiles might play a role as an intermediate state during the phase transition from the isotropic liquid state to *p*2*gg*, where pentagonal star‐shaped domains can frequently be observed during nucleation (see Figure S10, Supporting Information).

### 2.6 Local 2D‐Chirality

The reported 9‐hexagon lacks rotational symmetry and mirror symmetry perpendicular to the polygon plane. Therefore, this hexagon is chiral in 2D space and two enantiomers can be distinguished depending on the direction of the (2‐2‐1‐2‐1‐1) sequence (Figures 1h,3e). In the *p*2*gg* phase, dissymmetric pairs of enantiomeric *(P)*‐ and *(M)*‐9‐hexagons form an alternating 1:1 mixture (see Figure 3e).

### 2.7 Full sequence of Honeycombs of Series **1/*n***

**Figure 7:**
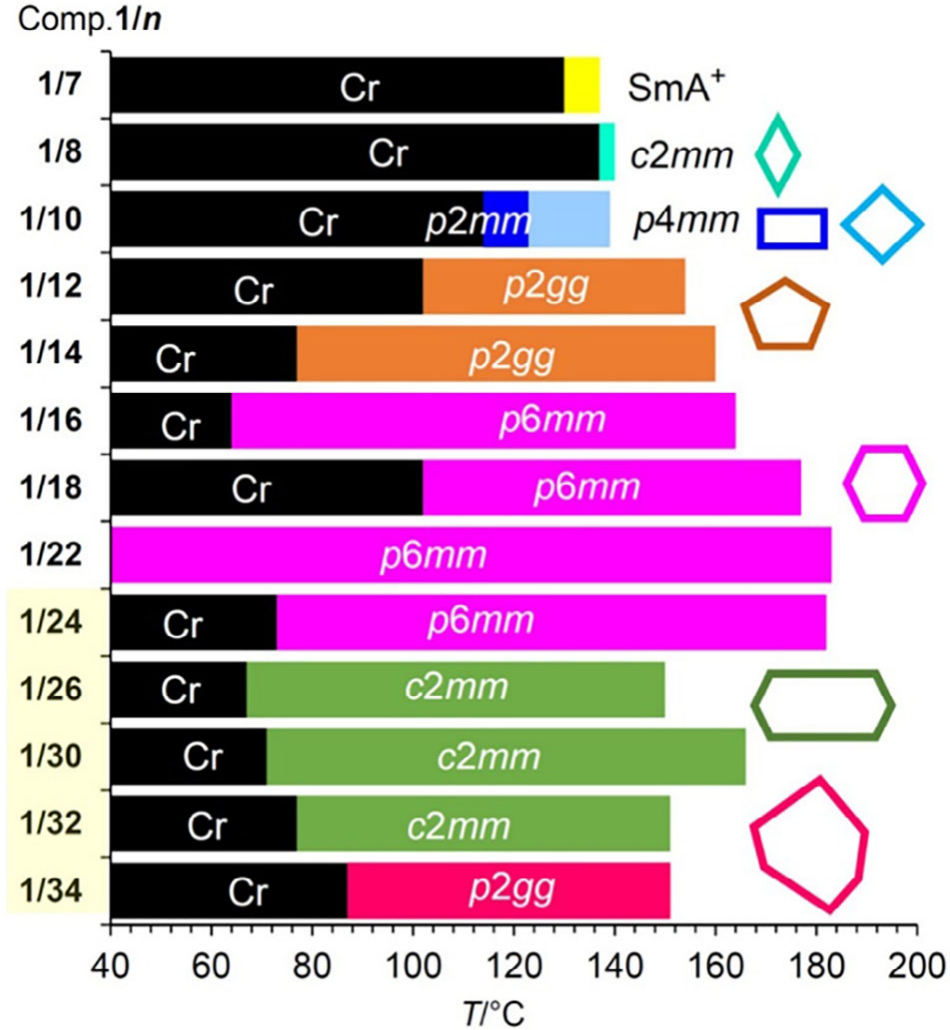
Development of LC honeycombs depending on alkyl chain length *n* in the series of compounds **1/*n*** as observed upon heating (for *n* = 7–22, see ref.[11]); the plane groups are given and the structure of each of the honeycombs is visualized by the shape of a single cell, shown at the right side. SmA^+^ is a distorted lamellar LC phase where the randomly distributed nano‐segregated alkyl chain domains provide the layer distortion.^[7a]^

The bar diagram in **Figure** **7** summarizes the complete phase sequence for the series of compounds **1/*n*** with *n* = 7–34. It impressively shows that based on the simple *p*‐terphenyl rod with two sticky end groups, eight different self‐assembled LC phases were realized exclusively by elongation of a *n*‐alkyl side chain. With exception of the lamellar phase of compound **1/7** (SmA^+^) all of them represent honeycombs formed by polygonal prismatic cells ranging from rhombic to hexagonal, including those with reduced symmetry (rhombuses, rectangles, (2‐1‐1)_2_ and (3‐1‐1)_2_‐hexagons) and two tilings with *p*2*gg* plane group formed by nonsymmetric 5‐pentagons^[^ ^11^, ^21^ ^]^ and nonsymmetric 9‐hexagons, respectively.

It is noted that no indication of an additional LC phase, possibly representing an intermediate 7‐hexagon honeycomb, could be detected at the transition from the 6‐hexagons to the 8‐hexagons. In the contact region between **1/24** (*p*6*mm*) and **1/26** (*c*2*mm*) a concentration gradient develops due to the diffusion of the compounds into each other, covering the intermediate chain volume range where such a phase could be expected. By POM investigation (see Figure S8, Supporting Information) only a direct transition *p*6*mm*‐*c*2*mm* is observed without any detectable induced LC phase.

### 2.8 Relations to Solid‐State Frameworks

**Figure 8:**
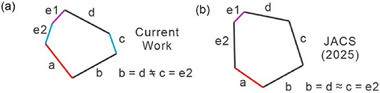
Comparison of the shapes of the hexagonal tiles a) reported in the current work with b) those recently reported for an solid‐state imine‐based COF,^[^ ^22^ ^]^ see Figure S18d,e (Supporting Information) for details.

Presently, there is only one other self‐assembled tiling by nonsymmetric hexagons, which was recently reported for the solid‐state structure of an imine‐based COF.^[^ ^22^ ^]^ Though it belongs to the same *type‐2* class of nonsymmetric hexagons^[^ ^18^ ^]^ it has a different shape of the hexagonal cells (for details of classification, see Figure S18d,e, Supporting Information). As shown in **Figure** **8**b it is formed by 4 long sides, one short side and one side having an intermediate length which is different from the (2‐2‐1‐2‐1‐1) sequence reported here (Figure 8a). The formation of the irregular COF hexagon tiling requires careful design of two different building blocks with well‐defined shapes fixing the node‐geometry, and a careful control of the developing imine bond conformation in the middle of the long sides during preparation. The tiling once prepared is fixed and well‐selected conditions are required to avoid contamination with other competing structures. With the help of system fluidity, our case doesn't require specific molecular orientations and inter‐molecular interaction directions.^[^ ^22^ ^]^ The smallest unit of our tiling is one single type of molecule, leading to the tiles with 9 identical molecules in the circumference being interconnected by dynamic hydrogen bonding networks which can adjust to any required junction‐geometry. That is why our hexagons are strictly self‐assembled thermodynamically stable equilibrium structures while the COFs are kinetically trapped. This provides the advantage that large uniform monodomains can easily be produced by alignment under the influence of external stimuli such as surfaces (see Figure 2c), shear fields (Figure 2d) and possibly also by electric or magnetic fields, while formation of larger single crystals is a problem in COF synthesis. Though the presence of the side‐chains removes hollow spaces for storage applications, the problems of network‐sliding and network interpenetration are also removed and non‐staggered side‐by‐side packing with honeycomb formation becomes intrinsically favored.

## 3 Conclusion

The first case of an irregular and non‐symmetric hexagonal honeycomb having an odd number of nine rod‐like units in the circumference of each prismatic cell has been discovered in a fluid self‐assembled and long‐range ordered liquid crystalline phase with periodicities in the nanometer range, and being stable over a temperature range of >60 K. Such system combining structural complexity with high molecular dynamics are promising for future design of stimuli‐responsive functional nano‐scale structures. Moreover, such fluid systems can serve as tool to find best solutions for uniform tilings of the Euclidian plane, similar to the Kelvin Problem in 3D space. If no solution can be found within the monohedral tessellations, then new honeycombs representing di‐ and multihedral^[^ ^8^ ^]^ and even quasiperiodic tiling patterns can be discovered.^[^ ^23^ ^]^ This could open new categories of designable material structures and LCs with potential exotic application properties.^[^ ^24^, ^25^ ^]^

## Conflict of Interest

The authors declare no conflict of interest

## Supporting information



Supporting Information

## Data Availability

The data that support the findings of this study are available in the supplementary material of this article.
