# Supplementary material for: A Self‐assembled Fluid Honeycomb of Nonsymmetric Hexagons Formed by Nine Molecules in the Circumference
Source: Small. 2025 Aug 23;21(40):e05094. doi: 10.1002/smll.202505094 (PMC12508708; doi:10.1002/smll.202505094)
Supplement: Supplementary file 1 — Supporting Information [file SMLL-21-e05094-s001.pdf]

NANO · MICRO
small

Supporting Information

for *Small*, DOI 10.1002/smll.202505094

A Self-assembled Fluid Honeycomb of Nonsymmetric Hexagons Formed by Nine Molecules in the Circumference

Christian Anders, Silvio Poppe, Yu Cao, Feng Liu and Carsten Tschierske**

Electronic Supporting Information

A self-assembled liquid crystal honeycomb of non-symmetric hexagons formed by nine molecules in the circumference

Christian Anders[a], Silvio Poppe[a], Yu Cao[b]*, Feng Liu[b], Carsten Tschierske[a]*

[a] Department of Chemistry, Martin Luther University Halle-Wittenberg, Kurt-Mothes-Str. 2, 06120 Halle, Germany

[b] Shannxi International Research Center for Soft Matter, State Key Laboratory for Mechanical Behavior of Materials, Xi'an Jiaotong University, Xi'an 710049, P. R. China.

Contents

S1. Synthesis and analytical data S2

S2. Methods S9

S3. Additional DSC data S11

S4. Additional POM textures S12

S5. Additional X-ray data S15

S6. Additional figures, notes and discussions S20

S7. Additional references S21

S1. Synthesis and analytical data

1.1 General

Synthesis of the compounds was performed according to Scheme 1 using the procedures described below. Unless otherwise noted, all starting materials were purchased from commercial sources and were used without further purification. 4,4''-Bis[(2,2-dimethyl-1,3-dioxolane-4-yl)methoxy]-p-terphenyl-2'-ol (**B**) was synthesized as reported in ref. [S1]. The long-chain alkyl bromides with n = 24 – 34 have been synthesized previously as reported in ref. [S2] Column chromatography was performed with silica gel 60 (63-200 µm, Fluka). Determination of structures and purity of intermediates and products was obtained by NMR spectroscopy (Agilent Technologies 400 MHz VNMRS, Agilent Technologies 600 MHz shielded VNMRS, all spectra were recorded at 27 °C with tetramethylsilane as internal standard). Microanalyses were performed using a CHNS-932 elemental analyzer (Leco). The water content of the analysed material was determined by Carl-Fischer titration as reported in ref. [S3]. HR-MS was performed with a Bruker HR-ESI-TOF in negative modus (3 mg/mL in THF/MeOH 9:1 solution with 10 µL aqu. LiCl solution, 4 mg LiCl/mL H_2O). The purity of all products was checked with thin layer chromatography (silica gel 60 F254, Merck). $CHCl_3$/EtOAc mixtures and $CHCl_3$/MeOH mixtures were used as eluents and the spots were detected by UV radiation, exposure to I_2 vapour or spraying with ammonium molybdate, $Ce(SO_4)_2/H_2SO_4$. All compounds represent racemic mixtures of diastereomers.

1.2 Synthesis of the acetonides C/*n*

A mixture of **B** (0.6 mmol) and the appropriate *n*-alkyl bromide (0.65 mmol), K_2CO_3 (165 mg, 1.2 mmol) and Bu_4NI (5 mg) in anhydrous DMF (30 mL) was stirred at 80 °C for 1 d. After cooling to room temperature, the reaction was poured into water (50 mL) and the aqueous layer was extracted with Et_2O (3x50 mL). The combined organic layers were washed with saturated aqu. LiCl, water and brine. After drying over anhydrous Na_2SO_4, filtration and evaporation of the solvent, the crude product was purified by column chromatography (silica gel, $CHCl_3$/EtOAc, 4/1 V/V).

4,4''-Bis[(2,2-dimethyl-1,3-dioxolane-4-yl)methoxy]-2'-tetracosanyloxy-p-terphenyl (C/24)

Yield: 273 mg (0.32 mmol, 54 %); colorless solid, $C_{54}H_{82}O_7$ 843.24 g/mol; ^{1}H-NMR ($CDCl_3$, *J*/Hz, 600 MHz) δ = 7.57 – 7.49 (m, 4H, Ar-*H*), 7.35 (d, ^3J(H,H) = 7.8 Hz, 1H, Ar-*H*), 7.18 (dd, ^3J(H,H) = 7.8, ^4J(H,H) = 1.7 Hz, 1H, Ar-*H*), 7.12 (d, ^4J(H,H) = 1.7 Hz, 1H, Ar-*H*), 7.02 – 6.93 (m, 4H, Ar-*H*), 4.55 – 4.47 (m, 2H, -O-C*H*), 4.23 – 4.16 (m, 2H, -O-CH_2-), 4.12 (dd, ^2J(H,H) = 9.4, ^3J(H,H) = 5.4 Hz, 2H, -O-CH_2-), 4.03 – 3.97 (m, 4H, -O-CH_2-), 3.96 – 3.91 (m, 2H, -O-CH_2-), 1.78 – 1.71 (m, 2H, -O-CH$_2$-CH_2-), 1.49 (s, 6H, - CH_3), 1.42 (s, 6H, - CH_3), 1.41 – 1.22 (m, 42H, - CH_2-), 0.88 (t, ^3J(H,H) = 7.0 Hz, 3H, - CH_3).

4,4''-Bis[(2,2-dimethyl-1,3-dioxolane-4-yl)methoxy]-2'-hexacosanyloxy-p-terphenyl (C/26)

Yield: 272 mg (0.31 mmol, 52 %); colorless solid, $C_{56}H_{86}O_7$ 871.30 g/mol; ^{1}H-NMR ($CDCl_3$, *J*/Hz, 400 MHz) δ = 7.59 – 7.49 (m, 4H, Ar-*H*), 7.35 (d, ^3J(H,H) = 7.9 Hz, 1H, Ar-*H*), 7.18 (dd, ^3J(H,H) = 7.8, ^4J(H,H) = 1.7

Hz, 1H, Ar-*H*), 7.12 (d, ^4J(H,H) = 1.7 Hz, 1H, Ar-*H*), 7.03 – 6.93 (m, 4H), 4.56 – 4.48 (m, 2H, -O-C*H*-), 4.23 – 4.17 (m, 2H, -O-CH_2-), 4.12 (dd, ^2J(H,H) = 9.5, ^3J(H,H) = 5.4 Hz, 2H, -O-CH_2-), 4.05 – 3.96 (m, 4H, -O-CH_2-), 3.94 (dd, ^2J(H,H) = 8.5, ^3J(H,H) = 5.8 Hz, 2H, -O-CH_2-), 2.06 – 1.98 (m, 2H, -O-CH$_2$-CH_2-), 1.79 – 1.70 (m, 2H, -O-CH$_2$-CH$_2$-CH_2-), 1.49 (s, 6H, - CH_3), 1.43 (s, 6H, - CH_3), 1.37 – 1.21 (m, 44H, - CH_2-), 0.89 (t, ^3J(H,H) = 6.8 Hz, 3H, - CH_3).

4,4''-Bis[(2,2-dimethyl-1,3-dioxolane-4-yl)methoxy]-2'-tricontanyloxy-p-terphenyl (C/30)

Yield: 240 mg (0.26 mmol, 43 %); colorless solid, $C_{60}H_{94}O_7$ 927.41 g/mol; ^{1}H-NMR (CDCl$_3$, *J*/Hz, 400 MHz) δ = 7.53 (m, 4H, Ar-*H*), 7.34 (d, ^3J(H,H) = 7.9 Hz, 1H, Ar-*H*), 7.18 (dd, ^3J(H,H) = 8.0 Hz, ^4J(H,H) = 1.5 Hz, 1H, Ar-*H*), 7.11 (d, 1H, ^4J(H,H) = 1.5 Hz, Ar-*H*), 6.97 (m 4H, Ar-*H*), 4.51 (m, 2H, -O-C*H*-), 4.19 (m, 2H, -O-CH_2-), 4.11 (dd, ^2J(H,H) = 9.3 Hz, ^3J(H,H) = 5.4 Hz, 2H, -O-CH_2-), 4.05-3.96 (m, 4H, -O-CH_2-), 3.92 (dd, ^2J(H,H) = 8.5 Hz, ^3J(H,H) = 5.6 Hz, 2H, -O-CH_2-), 1.74 (m, 2H, -O-CH$_2$-CH_2-), 1.48 (s, 6H, - CH_3), 1.42 (s, 6H, - CH_3), 1.41-1.16 (m, 54H, - CH_2-), 0.88 (t, ^3J(H,H) = 6.4 Hz, 3H, - CH$_3$).

4,4''-Bis[(2,2-dimethyl-1,3-dioxolane-4-yl)methoxy]-2'-dotricontanyloxy-p-terphenyl (C/32)

Yield: 283 mg (0.29 mmol, 49 %); colorless solid, $C_{62}H_{98}O_7$ 955.46 g/mol; ^{1}H-NMR (CDCl$_3$, *J*/Hz, 500 MHz) δ = 7.54 (m, 4H, Ar-*H*), 7.35 (d, ^3J(H,H) = 7.9 Hz, 1H, Ar-*H*), 7.18 (dd, ^3J(H,H) = 8.0 Hz, ^4J(H,H) = 1.5 Hz, 1H, Ar-*H*), 7.12 (d, 1H, ^4J(H,H) = 1.5 Hz, Ar-*H*), 6.98 (m 4H, Ar-*H*), 4.51 (m, 2H, -O-C*H*-), 4.19 (m, 2H, -O-CH_2-), 4.11 (dd, ^2J(H,H) = 9.3 Hz, ^3J(H,H) = 5.4 Hz, 2H, -O-CH_2-), 4.05-3.96 (m, 4H, -O-CH_2-), 3.93 (dd, ^2J(H,H) = 8.5 Hz, ^3J(H,H) = 5.6 Hz, 2H, -O-CH_2-), 1.74 (m, 2H, -O-CH$_2$-CH_2-), 1.48 (s, 6H, - CH_3), 1.42 (s, 6H, - CH_3), 1.41-1.17 (m, 58H, - CH_2-), 0.88 (t, ^3J(H,H) = 6.4 Hz, 3H, - CH$_3$).

4,4''-Bis[(2,2-dimethyl-1,3-dioxolane-4-yl)methoxy]-2'-tetratricontanyloxy-p-terphenyl (C/34)

Yield: 250 mg (0.25 mmol, 42 %); colorless solid, $C_{64}H_{102}O_7$ 983.51 g/mol; ^{1}H-NMR (CDCl$_3$, *J*/Hz, 402 MHz) δ = 7.58 – 7.48 (m, 4H, Ar-*H*), 7.35 (d, ^3J(H,H) = 7.8 Hz, 1H, Ar-*H*), 7.18 (dd, ^3J(H,H) = 7.9, ^4J(H,H) = 1.6 Hz, 1H, Ar-*H*), 7.12 (d, ^4J(H,H) = 1.8 Hz, 1H, Ar-*H*), 7.04 – 6.91 (m, 4H, Ar-*H*), 4.56 – 4.46 (m, 2H, -O-C*H*-), 4.23 – 4.17 (m, 2H, -O-CH_2-), 4.12 (dd, ^2J(H,H) = 9.4, ^3J(H,H) = 5.4 Hz, 2H, -O-CH_2-), 4.05 – 3.96 (m, 4H, -O-CH_2-), 3.96 – 3.91 (m, 2H, -O-CH_2-), 1.78 – 1.70 (m, 2H, -O-CH$_2$-CH_2-), 1.48 (s, 6H, -CH$_3$), 1.42 (s, 6H, -CH$_3$), 1.34 – 1.21 (m, 62H, - CH_2-), 0.88 (t, ^3J(H,H) = 6.7 Hz, 3H, - CH$_3$).

1.3 Compounds 1/*n*

The appropriate acetonide **B/*n*** (0.25 mmol) was dissolved in methanol (30 ml) and THF (30 ml) with pyridinium *p*-toluene sulfonate (PPTS, 50 mg) and stirred at 50 °C for 1 d. The progress of the reaction was monitored via TLC. After the consumption of all the starting material, the solvent was evaporated, and the crude product was purified by column chromatography (silica gel, CHCl$_3$/MeOH, 9/1 V/V) followed by recrystallization from the mixtures mentioned below.

4,4''-Bis(2,3-dihydroxypropyloxy)-2'-tetracosanyloxy-*p*-terphenyl (1/24)

Purified by crystallization from MeOH/THF; yield 139 mg (0.18 mmol, 72 %) colorless solid; ^{1}H-NMR ($CDCl_3$, *J*/Hz, 402 MHz) δ = 7.53 (m, 4H, Ar-*H*), 7.33 (d, ^3J(H,H) = 7.8 Hz, 1H, Ar-*H*), 7.16 (dd, ^3J(H,H) = 8.0 Hz, ^4J(H,H) = 1.5 Hz, 1H, Ar-*H*), 7.10 (d, ^4J(H,H) = 1.5 Hz, 1H, Ar-*H*), 6.96 (m, 4H, Ar-*H*), 4.16-4.05 (m, 6H, -C*H*-OH, -CH_2-OH), 4.00 (t, ^3J(H,H) = 6.5 Hz, 2H, -O-CH_2-), 3.92 – 3.83 (m, 2H, ArO-C$H_A$$H_B$), 3.76 (dd, ^2J(H,H) = 11.5 Hz, ^3J(H,H) = 5.2 Hz, 2H, ArO-C$H_A$$H_B$), 1.73 (m, 2H, -O-$CH_2$-C$H_2$-), 1.38 (m, 2H, -O-$CH_2$-$CH_2$-C$H_2$-), 1.32-1.16 (m, 40H, -CH_2-), 0.86 (t, ^3J(H,H) = 6.9 Hz, 3H, CH_3). ^{13}C-NMR ($CDCl_3$, 101 MHz) δ = 158.0, 157.3, 156.3 (Ar-O), 140.8, 134.3, 131.4 (Ar_{quart}), 130.8, 130.7 (Ar-H), 128.8 (Ar_{quart}), 128.2, 119.2, 114.8, 113.9, 111.1 (Ar-H), 70.3, 69.3, 69.2, 68.5, 63.7, 63.6 (CHO, CH_2O), 31.8 (CH_2), 29.7, 29.7 (multiple CH_2), 29.6, 29.6, 29.6, 29.3, 29.3, 29.2, 26.1, 22.7 (CH_2), 14.1 (CH_3)*;* HR-MS (m/z): [M]+Cl^- calcd. for $C_{48}H_{74}O_7Cl$, 797.5118; found: 797.5128, EA: calc. for $C_{48}H_{74}O_7$: C 75.55 %, H 9.77 %, found: C 75.57 %, H 9.69 %.

4,4''-Bis(2,3-dihydroxypropyloxy)-2'-hexacosanyloxy-*p*-terphenyl (1/26)

Purified by crystallization from MeOH/THF; yield 152 mg (0.19 mmol, 77 %) colorless solid; ^{1}H-NMR (pyridine-d_5, *J*/Hz, 500 MHz) δ = 7.74 (m, 4H, Ar-*H*), 7.51 (d, ^3J(H,H) = 7.8 Hz, 1H, Ar-*H*), 7.40 (d, ^4J(H,H) = 1.5 Hz, 1H, Ar-*H*), 7.37 (dd, ^3J(H,H) = 8.0 Hz, ^4J(H,H) = 1.5 Hz, 1H, Ar-*H*), 6.21 (m, 4H, Ar-*H*), 4.6-4.4 (m, 6H, -C*H*-OH, -CH_2-OH), 4.27-4.15 (m, 4H, ArO-CH_2), 4.04 (t, ^3J(H,H) = 6.4 Hz, 2H, ArO-CH_2-R), 1.73 (m, 2H, -O-CH_2-CH_2-), 1.38 (m, 2H, -O-CH_2-CH_2-CH_2-), 1.4-1.2 (m, 44H, -CH_2-), 0.84 (t, ^3J(H,H) = 6.8 Hz, 3H, CH_3). ^{13}C-NMR (pyridine-d_5, 126 MHz) δ = 159.4, 158.7, 156.7 (Ar-O), 141.1, 133.5, 131.1 (Ar_{quart}), 131.1, 130.9 (Ar-H), 129.1 (Ar_{quart}), 128.2, 119.3, 115.3, 114.4, 111.2 (Ar-H), 71.3, 70.8, 70.7, 68.4, 64.2, 64.1 (CHO, CH_2O), 31.9, 31.9, 31.7 (CH_2), 29.8, 29.7 (multiple CH_2), 29.7, 29.6, 29.6, 29.5, 29.4, 29.3, 27.3, 26.2, 22.7, 22.7(CH_2), 14.0 (CH_3)*;* HR-MS (m/z): [M]+Cl^- calcd. for $C_{50}H_{78}O_7Cl$, 825.5431; found: 825.5446; EA: calc. for $C_{50}H_{78}O_7$: C 75.91 %, H 9.94 %, found: C 75.65 %, H 10.22 %.

4,4''-Bis(2,3-dihydroxypropyloxy)-2'-tricontanyloxy-*p*-terphenyl (1/30)

Purified by crystallization from MeOH/THF; yield 180 mg (0.21 mmol, 85 %) colorless solid; ^{1}H-NMR (pyridine-d_5, *J*/Hz, 400 MHz) δ = 7.85-7.75 (m, 4H, Ar-*H*), 7.56 (d, ^3J(H,H) = 7.8 Hz, 1H, Ar-*H*), 7.45 (d, ^4J(H,H) = 1.3 Hz, 1H, Ar-*H*), 7.42 (dd, ^3J(H,H) = 7.9 Hz, ^4J(H,H) = 1.5 Hz, 1H, Ar-*H*), 7.31-7.23 (m, 4H, Ar-*H*), 6.91 (broad, 2H, OH) 6.52 (broad, 2H, OH), 4.68-4.44 (m, 6H, -C*H*-OH, -CH_2-OH), 4.34-4.19 (m 4H, ArO-CH_2-), 4.09 (t, ^3J(H,H) = 6.4 Hz, 2H, R-CH_2-OAr), 1.85-1.70 (m, 2H, -O-CH_2-CH_2-), 1.54-1.40 (m, 2H, -O-CH_2-CH_2-CH_2-), 1.40-1.08 (m, 52H, -CH_2-), 0.88 (t, ^3J(H,H) = 6.8 Hz, 3H, CH_3). ^{13}C-NMR (pyridine-d_5, 126 MHz) δ = 159.4, 158.7, 156.7 (Ar-O), 141.1, 133.5, 131.1 (Ar_{quart}), 131.1, 130.9 (Ar-H), 129.1 (Ar_{quart}), 128.3, 119.3, 115.3, 114.4, 111.3 (Ar-H), 71.3, 70.8, 70.7, 68.5, 64.2, 64.1 (CHO, CH_2O), 31.9 (CH_2), 29.8, 29.8 (multiple CH_2), 29.7, 29.7, 29.7, 29.4, 29.3, 26.2, 22.7 (CH_2), 14.0 (CH_3)*;* HR-MS (m/z): [M]+Cl^- calcd. for $C_{54}H_{86}O_7Cl$, 881.6057; found: 881.6084; EA: calc. for $C_{54}H_{86}O_7$: C 76.55 %, H 10.23 %, found: C 76.28 %, H 10.05 %.

4,4''-Bis(2,3-dihydroxypropyloxy)-2'-dotricontanyloxy-*p*-terphenyl (1/32)

Purified by crystallization from MeOH/THF; yield 173 mg (0.20 mmol, 79 %) colorless solid; ^{1}H-NMR (pyridine-d_5, *J*/Hz, 402 MHz) δ = 7.77 (m, 4H, Ar-*H*), 7.53 (d, ^3J(H,H) = 7.8 Hz, 1H, Ar-*H*), 7.42 (d, ^4J(H,H) = 1.5 Hz, 1H, Ar-*H*), 7.40 (dd, ^3J(H,H) = 8.0 Hz, ^4J(H,H) = 1.5 Hz, 1H, Ar-*H*), 7.24 (m, 4H, Ar-*H*), 4.63-4.43 (m, 6H, -C*H*-OH, -CH_2-OH), 4.28-4.19 (m, 4H, ArO-CH_2), 4.06 (t, ^3J(H,H) = 6.4 Hz, 2H, ArO-CH_2-R), 1.75

(m, 2H, -O-CH_2-CH_2-), 1.44 (m, 2H, -O-CH_2-CH_2-CH_2-), 1.38-1.20 (m, 56H, -CH_2-), 0.86 (t, ^3J(H,H) = 7.1 Hz, 3H, CH_3). ^{13}C-NMR (pyridine-d_5, 101 MHz) δ = 160.9.0, 160.2, 158.2 (Ar-O), 142.6, 136.0, 132.6 (Ar_{quart}), 132.6, 132.4 (Ar-H), 130.6 (Ar_{quart}), 129.8, 120.8, 116.8, 115.9, 112.7 (Ar-H), 72.8, 72.3, 72.2, 70.0, 65.7 (CHO, CH_2O), 33.4 (CH_2), 31.3, 31.3 (multiple CH_2), 31.2, 31.1, 30.9, 30.8, 27.7, 24.2, (CH_2), 15.5 (CH_3); HR-MS (m/z): [M]+Cl^- calcd. for $C_{56}H_{90}O_7Cl$, 881.68419; found: 881.68372; EA: calc. for $C_{56}H_{90}O_7 \cdot H_2O$: C 75.29 %, H 10.38 %, found: C 75.50 %, H 10.33 %.

4,4''-Bis(2,3-dihydroxypropyloxy)-2'-tetratricontanyloxy-*p*-terphenyl (1/34)

Purified by crystallization from MeOH/THF; yield 147 mg (0.16 mmol, 65 %) colorless solid; ^{1}H-NMR (pyridine-d_5, *J*/Hz, 402 MHz) δ = 7.76 (m, 4H, Ar-*H*), 7.51 (d, ^3J(H,H) = 7.9 Hz, 1H, Ar-*H*), 7.40 (s, 1H, Ar-*H*), 7.37 (d, ^3J(H,H) = 8.0 Hz, 1H, Ar-*H*), 7.22 (m, 4H, Ar-*H*), 4.61-4.42 (m, 6H, -C*H*-OH, -CH_2-OH), 4.26-4.19 (m, 4H, ArO-CH_2), 4.04 (t, ^3J(H,H) = 6.4 Hz, 2H, ArO-CH_2-R), 1.73 (m, 2H, -O-CH_2-CH_2-), 1.42 (m, 2H, -O-CH_2-CH_2-CH_2-), 1.34-1.16 (m, 60H, -CH_2-), 0.84 (t, ^3J(H,H) = 6.6 Hz, 3H, CH_3). ^{13}C-NMR (pyridine-d_5, 101 MHz) δ = 159.4, 158.7, 156.7 (Ar-O), 141.1, 133.5, 131.1 (Ar_{quart}), 131.1, 130.9 (Ar-H), 129.1 (Ar_{quart}), 128.3, 119.3, 115.3, 114.4, 111.3 (Ar-H), 71.3, 70.8, 70.7, 68.4, 64.2, 64.1 (CHO, CH_2O), 31.9 (CH_2), 29.8, 29.7 (multiple CH_2), 29.7, 29.6, 29.4, 29.3, 26.2, 22.7 (CH_2), 14.0 (CH_3); HR-MS (m/z): [M]+Cl^- calcd. for $C_{58}H_{94}O_7Cl$, 937.6683; found: 937.6702; EA: calc. for $C_{58}H_{94}O_7$: C 77.12 %, H 10.49 %, found: C 76.96 %, H 10.18 %.

1.4 Representative NMR spectra

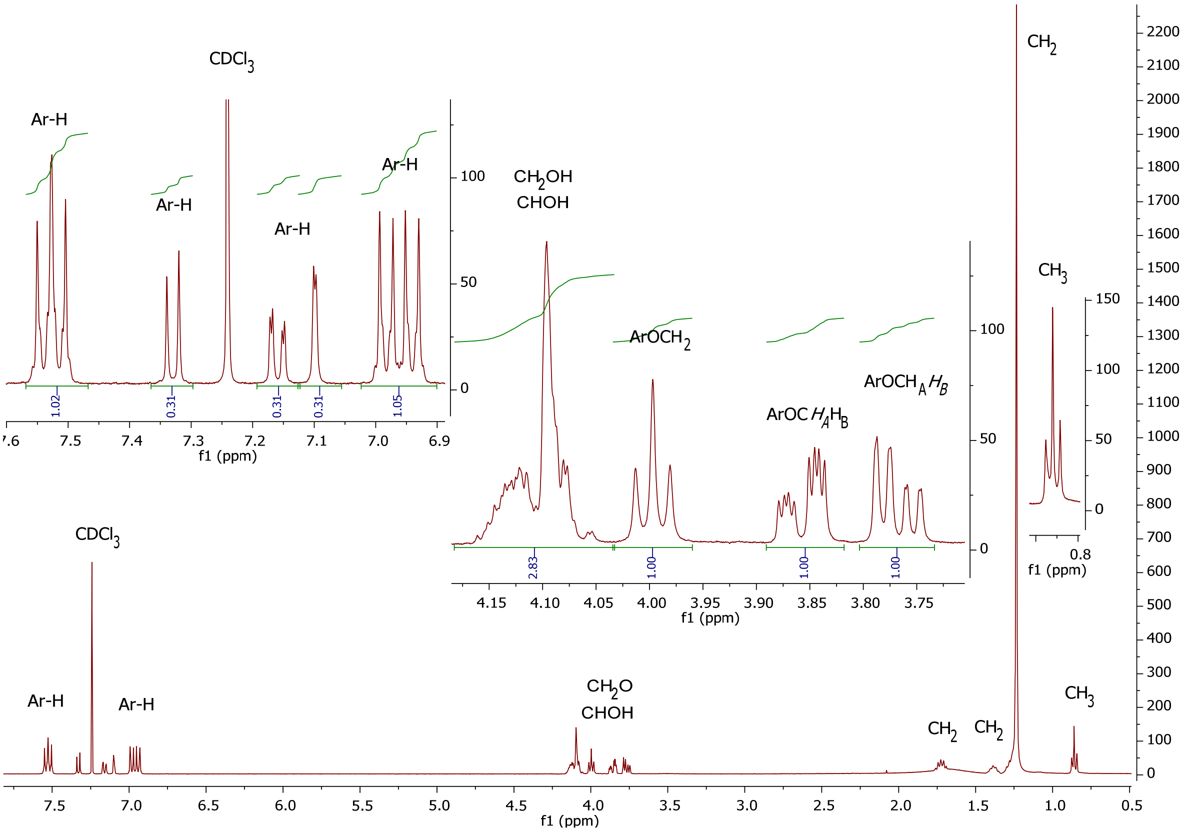

Figure S1. ^{1}H-NMR spectrum of **1/24** ($CDCl_3$, 402 MHz, 27°C)

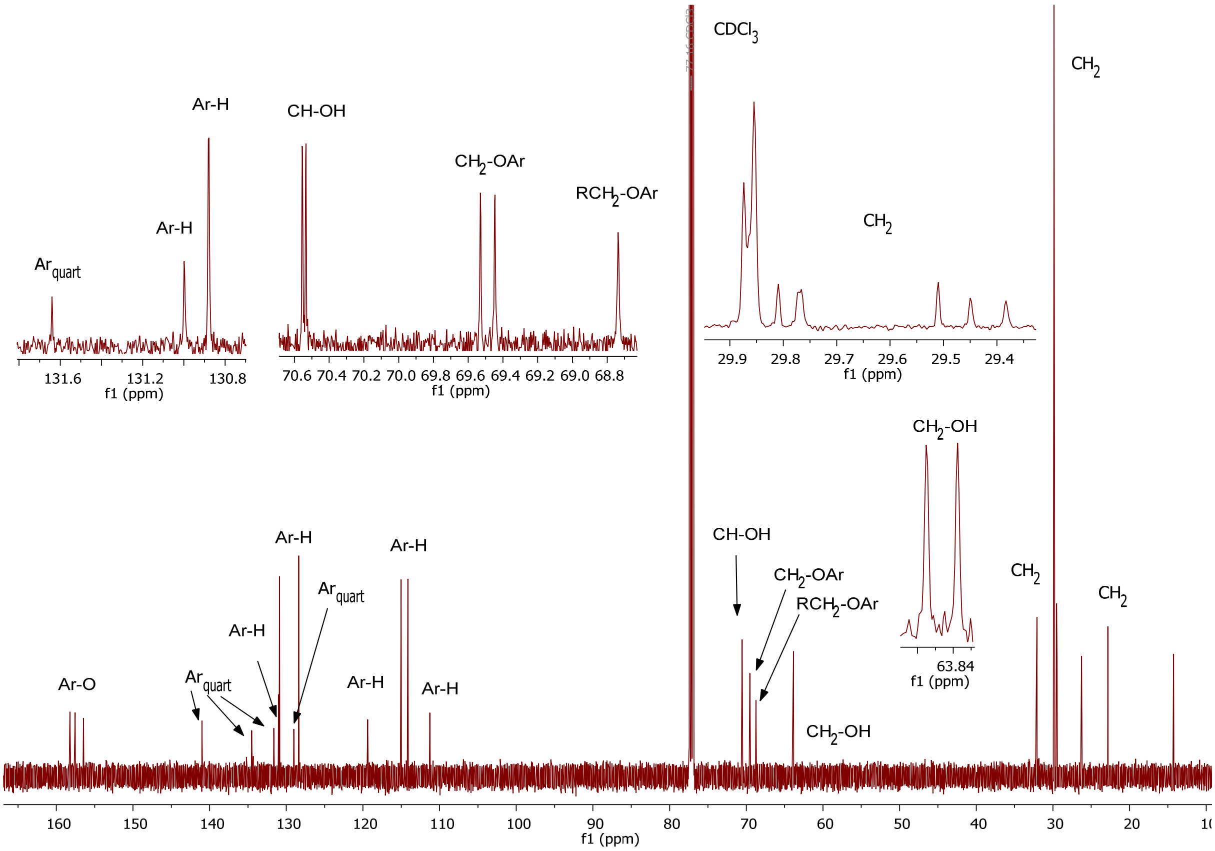

Figure S2. ^{13}C-NMR spectrum of **1/24** ($CDCl_3$, 101 MHz, 27°C)

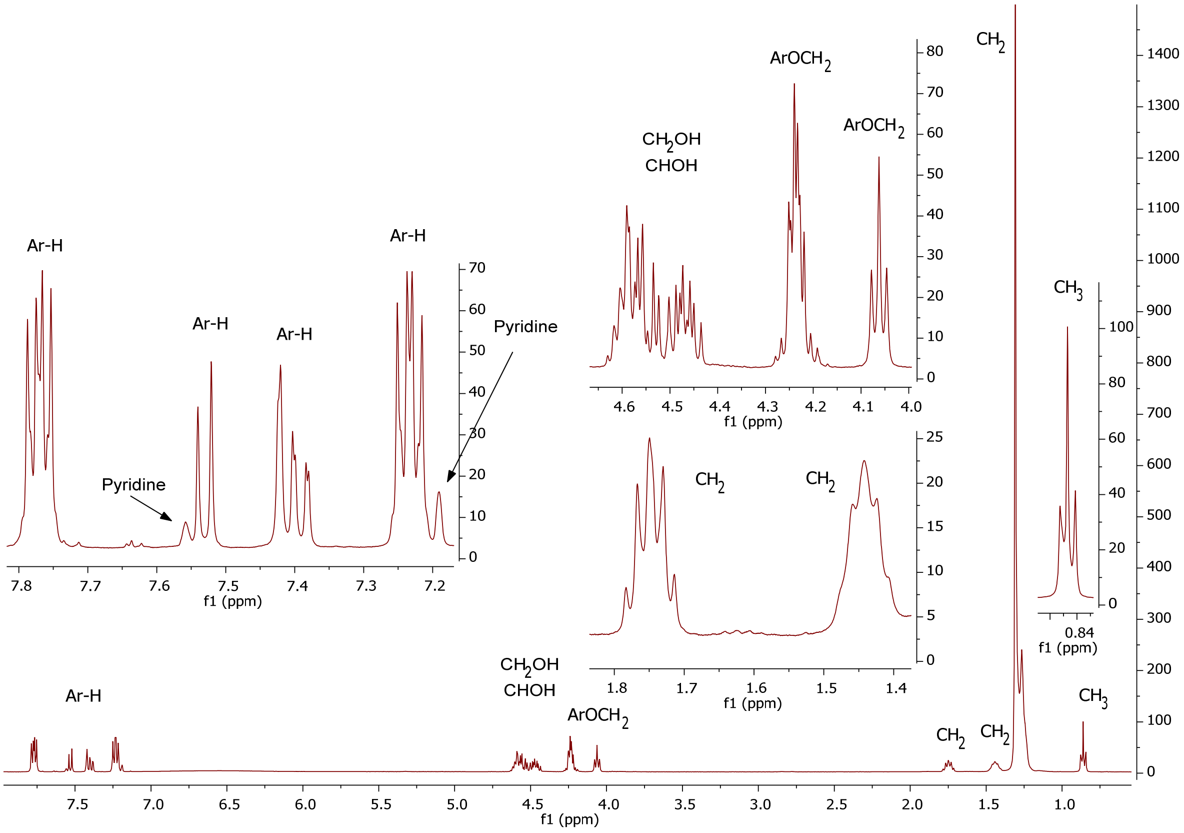

Figure S3. ^{1}H-NMR spectrum of **1/32** (pyridine-d5, 402 MHz, 27°C)

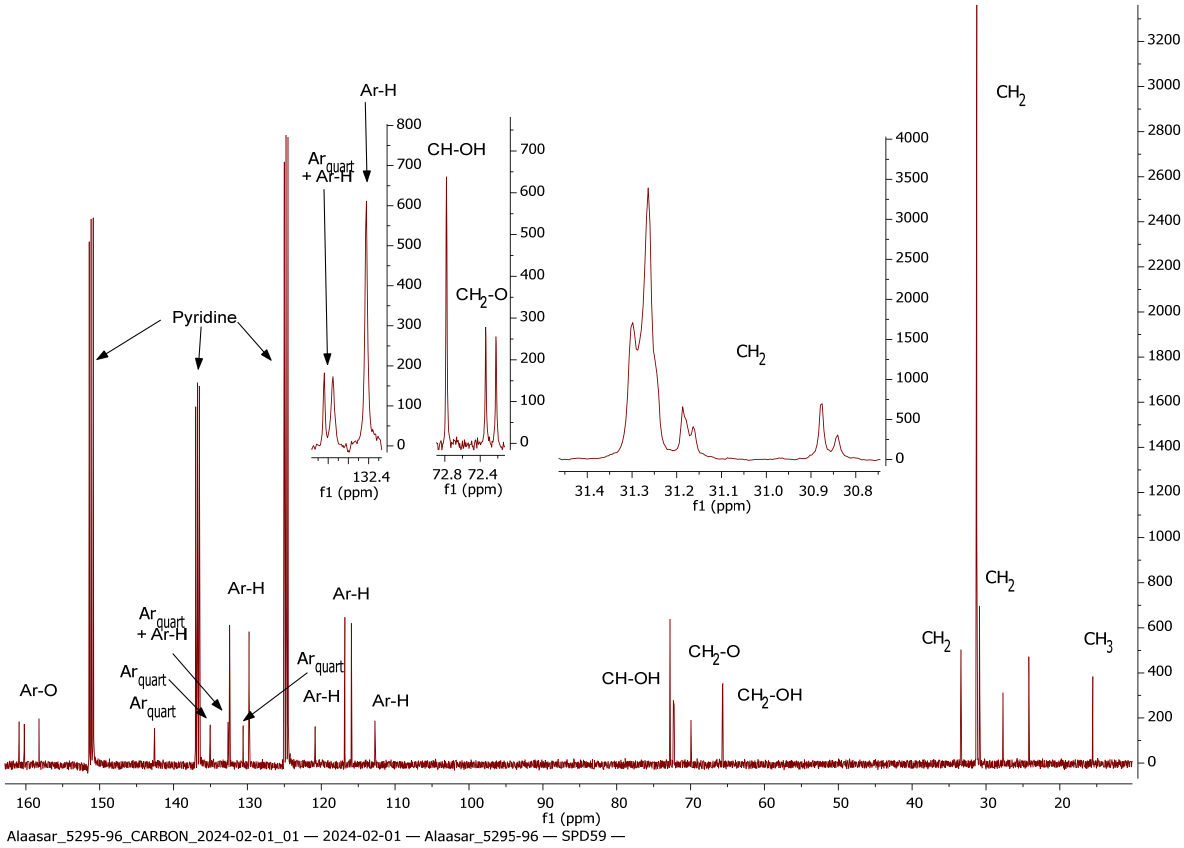

Figure S4. ^{13}C-NMR spectrum of **1/32** (pyridine-d5, 101 MHz, 27°C)

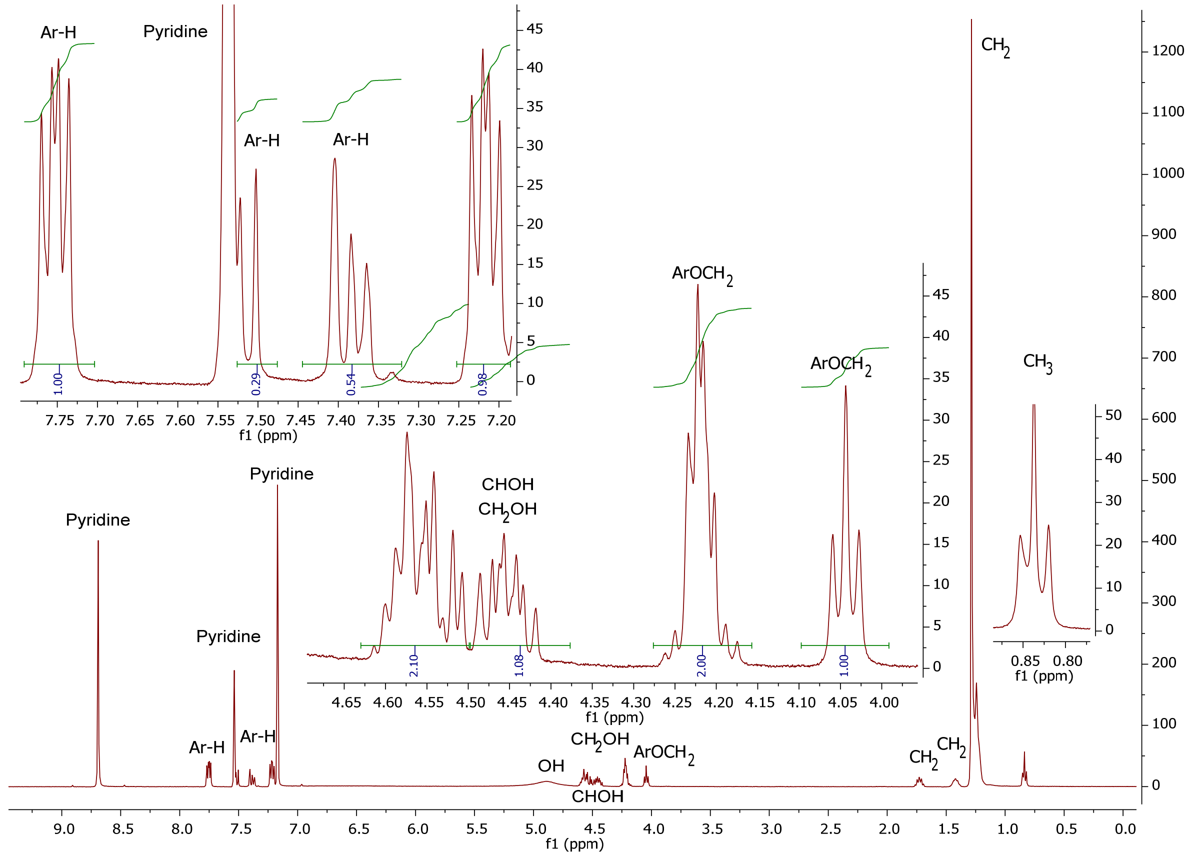

Figure S5. ^{1}H-NMR spectrum of **1/34** (pyridine-d₅, 402 MHz, 27°C)

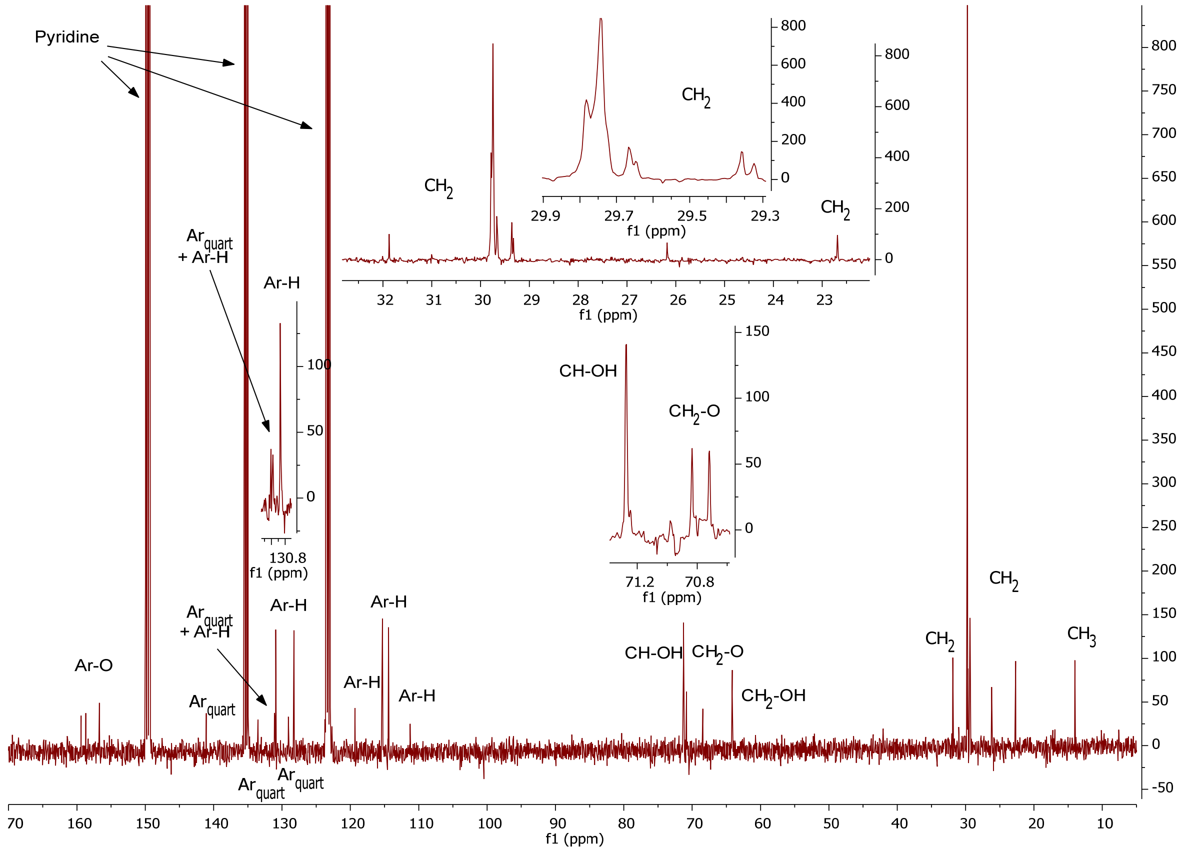

Figure S6. ^{13}C-NMR spectrum of **1/34** (pyridine-d₅, 101 MHz, 27°C).

S2. Methods

Sample preparation. Open samples of the compounds ***1/n*** were heated to 170 °C for 20 seconds to remove all water traces and were immediately sealed just before investigation. This procedure allows complete removal of water traces. [S3 ,S4]

Polarizing Optical Microscopy (POM). Transition temperatures were measured using a Mettler FP-82 HT hot stage and control unit in conjunction with a Leica DMRXP polarizing microscope (Leica Microsystems GmbH, Wetzlar, Germany). Textures of the liquid crystalline mesophases were recorded with a Leica MC120 HD camera (Leica Microsystems GmbH, Wetzlar, Germany) between non-treated microscopy glass plates.

DSC measurements. DSC-thermograms were recorded on a DSC-7 (Perkin-Elmer GmbH & Co. KG, Überlingen, Germany) and a DSC-8000 (Perkin Elmer) in 30 µL Al-pans with heating and cooling rates of 10 K/min.

X-ray scattering. - X-ray investigations were carried out at Cu Kα line (λ = 1.54 Å) using a standard Coolidge tube source with a Ni-filter. Aligned samples were obtained on a glass plate on a temperature-controlled heating stage. Alignment was achieved upon slow cooling (rate: 1 K·min^{-1} – 0.01 K·min^{-1}) of a small droplet of the sample and takes place at the sample–glass or at the sample–air interface. The samples were held on a temperature-controlled heating stage and the diffraction patterns were recorded with a Vantec 500 area detector (Bruker AXS, Karlsruhe); the exposure time was 3 min for WAXS and 5 min for SAXS. The distance between the sample and the detector was 9.0 cm (WAXS) or 26.8 cm (SAXS), and the beam was parallel to the substrate.

Synchrotron X-ray diffraction and electron density reconstruction. - High-resolution small-angle powder diffraction experiments were recorded on Beamline I22 at Diamond Light Source and Beamline BL16B1 at Shanghai Synchrotron Radiation Facility, SSRF. Samples were held in evacuated 1 mm capillaries. A modified Linkam hot stage with a thermal stability within 0.2 ºC was used, with a hole for the capillary drilled through the silver heating block and mica windows attached to it on each side. A MarCCD detector was used. *q* calibration and linearization were verified using several orders of layer reflections from silver behemate and a series of *n*-alkanes. The measurement of the positions and intensities of the diffraction peaks is carried out using Galactic PeakSolve™ program, where experimental diffractograms are fitted using Gaussian shaped peaks. The diffraction peaks are indexed on the basis of their peak positions, and the lattice parameters and the space groups are subsequently determined. Once the diffraction intensities are measured and the corresponding plane group determined, electron density maps can be reconstructed, on the basis of the general formula

$$E(xy) = \Sigma_{hk} F(hk) \exp[i2\pi(hx+ky)] \qquad \text{(Eqn. 1)}$$

2.

Here $F(hk)$ is the structure factor of a diffraction peak with index (hk). It is normally a complex number and the experimentally observed diffraction intensity

$$I(hk) = K\cdot F(hk)\cdot F^*(hk) = K\cdot|F(hk)|^2 \qquad \text{(Eqn. 2)}$$

3.

Here K is a constant related to the sample volume, incident beam intensity etc. In this paper we are only interested in the relative electron densities, hence this constant is simply taken to be 1. Thus the electron density

$$E(xy) = \Sigma_{hk}\mathrm{sqrt}[I(hk)]\ \exp[i2\pi(hx+ky)+\phi_{hk}] \qquad \text{(Eqn. 3)}$$

As the observed diffraction intensity $I(hk)$ is only related to the amplitude of the structure factor $|F(hk)|$, the information about the phase of $F(hk)$, ϕ_{hk}, cannot be determined directly from experiment. However, the problem is much simplified when the structure of the ordered phase is centrosymmetric, and hence the structure factor $F(hk)$ is always real and ϕ_{hk} is either 0 or π.

This makes it possible for a trial-and-error approach, where candidate electron density maps are reconstructed for all possible phase combinations, and the "correct" phase combination is then selected on the merit of the maps, helped by prior physical and chemical knowledge of the system. This is especially useful for the study of nanostructures, where normally only a limited number of diffraction peaks are observed.

GISAXS experiments were carried out on station BM28 (XMaS line) at ESRF. Thin films were prepared from the melt on a silicon wafer. The thin film coated 5 x 5 mm^2 Si plates were placed on top of a custom-built heater, which was then mounted on a six-circle goniometer. A MarCCD 165 detector at ESRF was used. The sample enclosure and the beam pipe were flushed with helium.

The d*V*/d*r* calculation. d*V*/d*r* curves are used to evaluate the relate the aliphatic tails with overall space filling in the lattice. Three types of tilings are included, see Fig S20. All phases involved are columnar phases, *r* represents the radius of circles centered at the middle point of each wall and *V* stands for the volume of the formed cylinder. Considering the 2D symmetry, we took cross-section area as *V* in the paper. Each lattice is divided into small squares of 0.01*0.01 nm^2 and numerical method was applied to calculate the volume increment with increasing *r*.

S3. Additional DSC traces

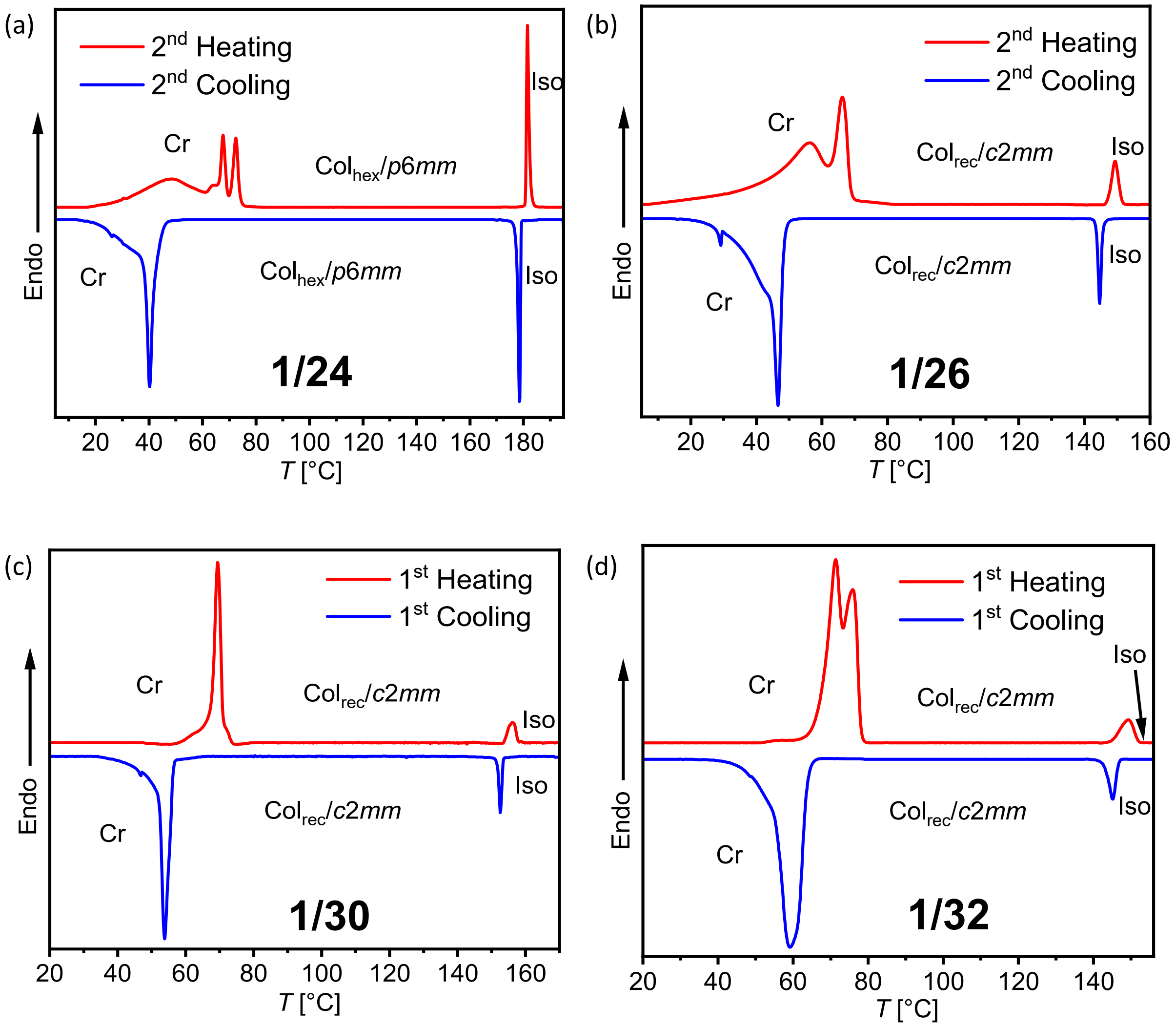

Figure S7. DSC heating (top) and cooling scans (bottom) of compounds **1/24-1/32** (10 K min^{-1}).

S4. Additional POM-textures

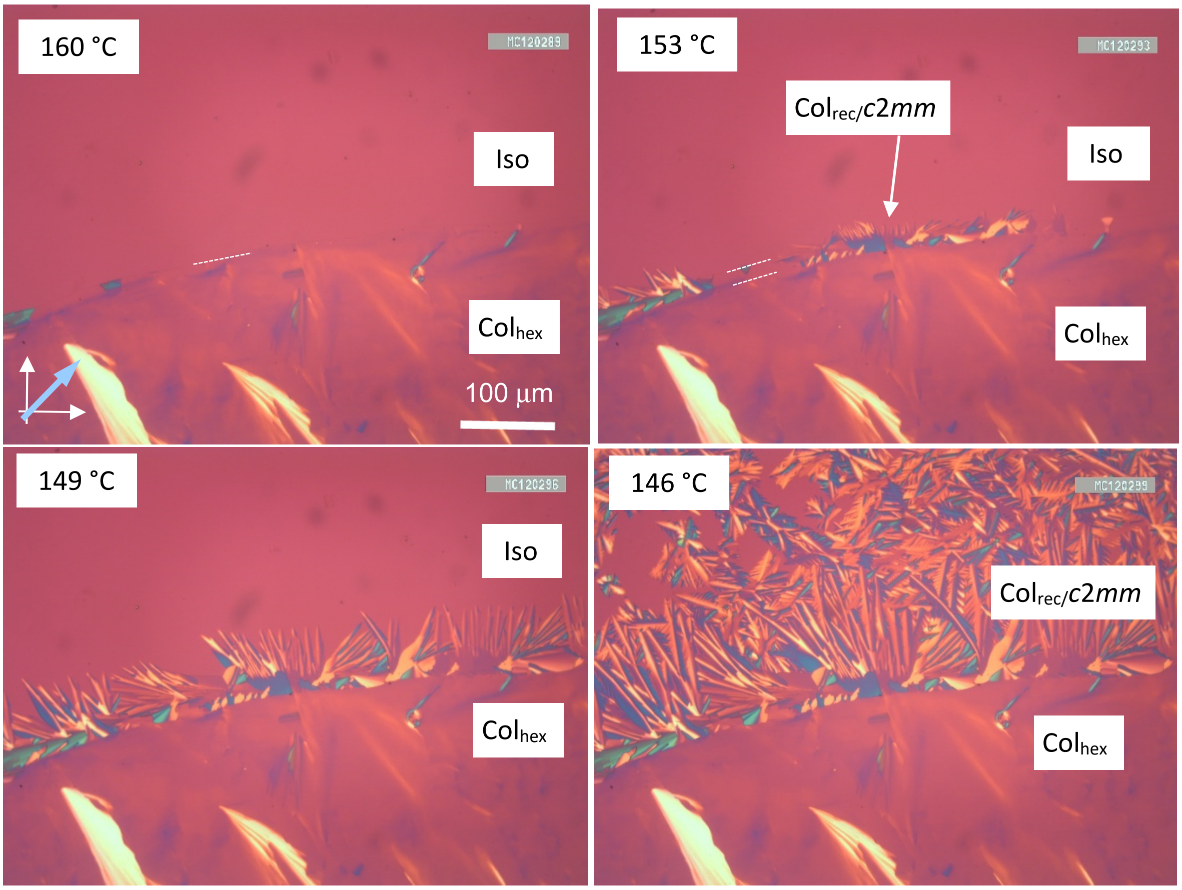

Figure S8. Contact region between the Col$_{hex}$ phase of **1/24** (bottom) and **1/26** (top) as observed on cooling between crossed polarizers and with additional λ-plate (indicatrix direction shown as blue arrow); the development of the Col$_{rec}$/*c*2*mm* phase starts at 154 °C in a small stripe of the in the Col$_{hex}$ phase in the contact region (between the dotted white lines) and at 153 °C already grows into the Iso phase of **1/26**. Interestingly, no indication of any monohedral tiling by 7-numbered polygons is found at the transition from 6-hexagon to 8-hexagon. In the contact region between **1/24** and **1/26** no additional induced phase can be detected between *p*6*mm* and *c*2*mm* by means of polarizing optical microscopy. The reason might be the restrictions provided by the fixed length of the building blocks of the polygons which provides no solution for a monohedral tiling of 7-hexagons (or 7-pentagons) without gaps.

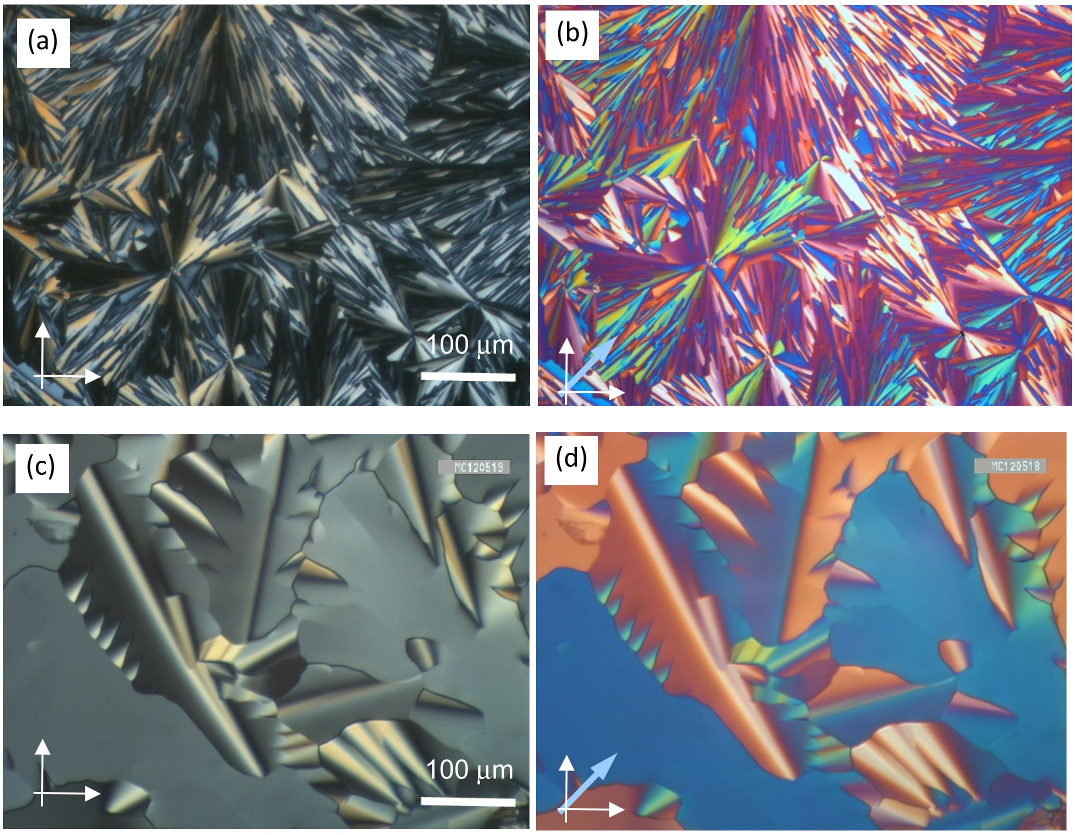

Figure S9. (a) POM textures of the $\mathrm{Col_{rec}}/c2mm$ phases of (a, b) **1/30** at 140 °C and (c, d) **1/32** at 149 °C on cooling, in (b, d) with λ-plate.

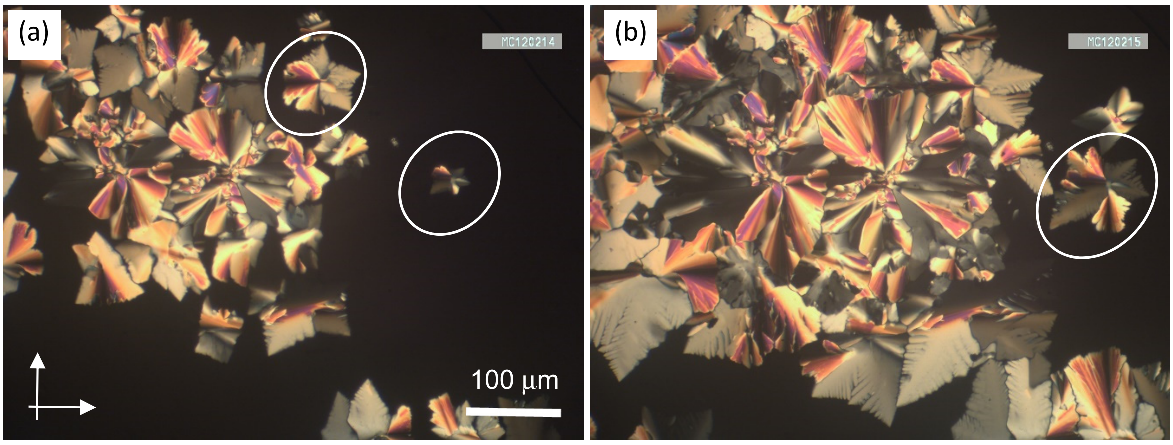

Figure S10. Growth of the Col_{rec}/*p*2*gg* phase of **1/34** from Iso at *T* = 149 °C. The growth of some domains as pentagonal stars might indicate that growth in homeotropic alignment starts with pentagons (low birefringent segments) which then deform into the hexagons upon further growth, thus inducing a stress, leading to a transition to planar alignment in the highly birefringent segments.

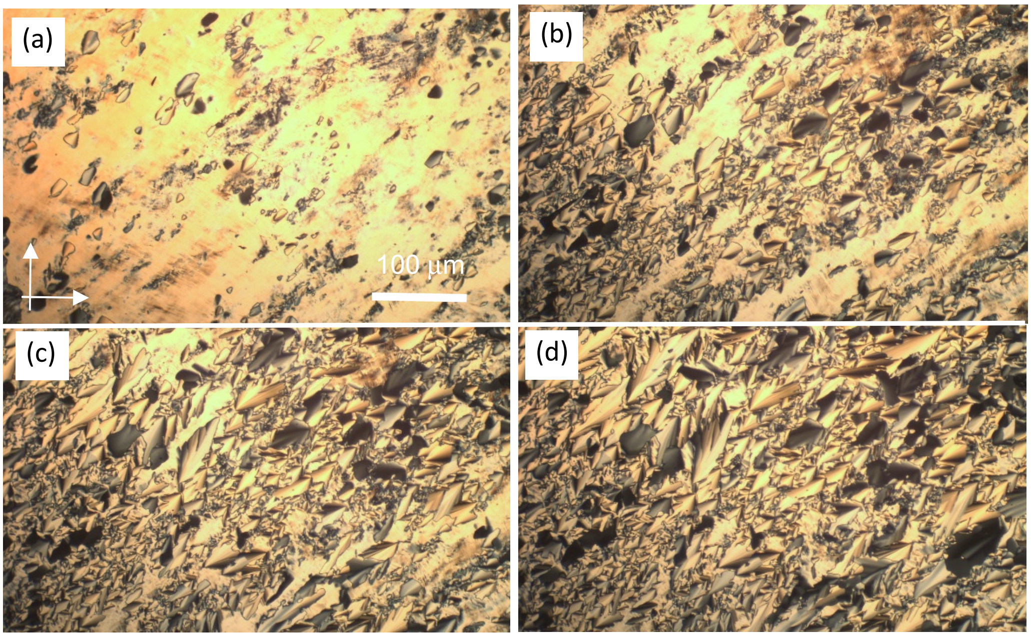

Figure S11. Reformation of the fan-like texture of the Col_{rec}/*p*2*gg* phase of **1/34** from a sheared sample at *T* = 143 °C after (a) 2 min, (b) 5 min, (c) 15 min and (d) 30 min, confirming the fluidity and capability of liquid state self-assembly.

S5. Additional XRD data

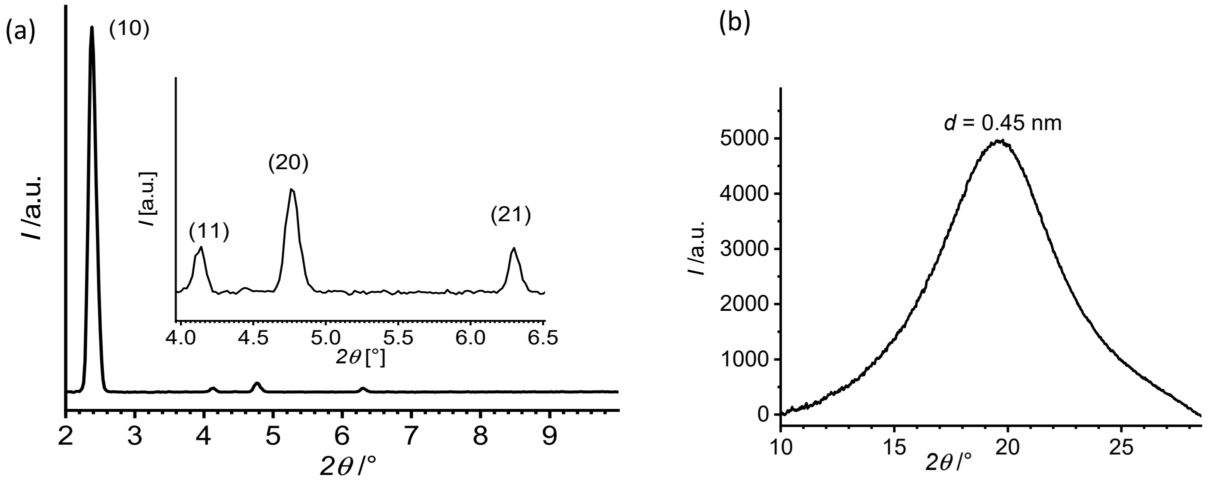

Figure S12. (a) SAXS and (b) WAXS patterns of the Col_{hex}/*p*6*mm* phase of **1/24** at *T* = 140 °C.

Table S1. Numerical XRD data of the Col_{hex}/*p*6*mm* phase of **1/24** at *T* = 140 °C

**2*θ* /°**	***d*$_{obs.}$ /nm**	***d*$_{calc.}$ /nm**	**Δ**	***hk***
2.387	3.701	3.701	0.00	10
4.130	2.139	2.137	0.00	11
4.769	1.853	1.851	0.00	20
6.302	1.402	1.399	0.00	21
19.563	0.454			

Col_{hex}/*p*6*mm*: a_{hex} = 4.28 nm

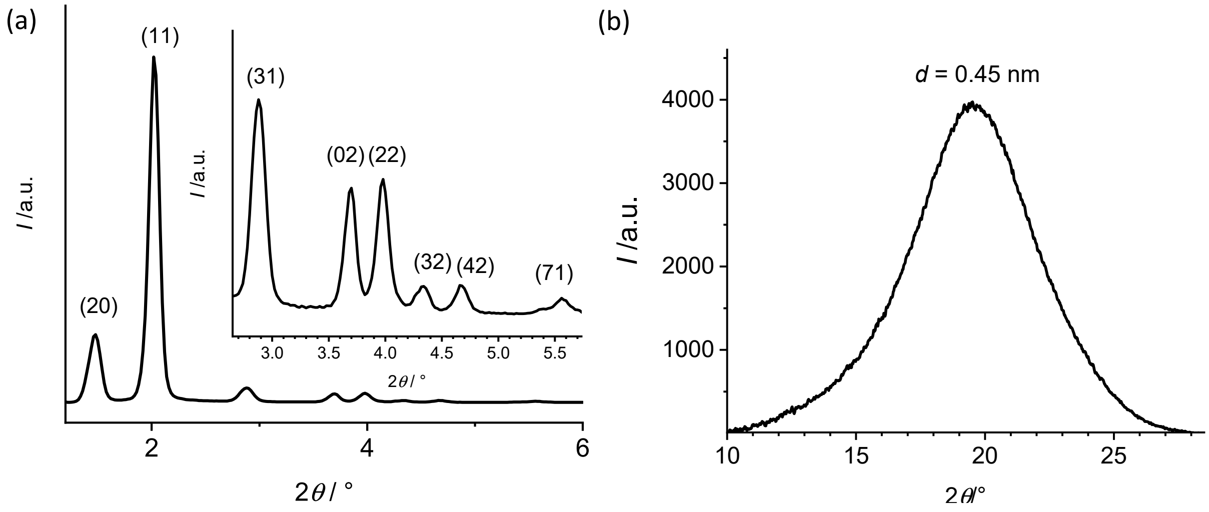

Figure S13. (a) SAXS and (b) WAXS patterns of the Col_{rec}/*c*2*mm* phase of **1/26** at *T* = 110 °C.

Table S2. Numerical XRD data of the Col_{rec}/*c*2*mm* phase of **1/26** at *T* = 110 °C.

**2*θ* /°**	***d*obs. /nm**	***d*calc. /nm**	**Δ**	***hk***
1.475	5.989	5.989	0.00	20
2.02	4.373	4.373	0.00	11
2.894	3.053	3.042	0.01	31
3.654	2.393	2.349	0.04	02
3.973	2.221	2.186	0.04	22
4.339	2.042	2.024	0.02	32
4.669	1.891	1.848	0.04	42
5.382	1.642	1.677	0.04	52
5.566	1.588	1.566 / 1.608	0.02	03 / 71
19.269	0.461			

Col_{rec}/*c*2*mm*: a_{rec} = 11.98 nm; b_{rec} = 4.70 nm

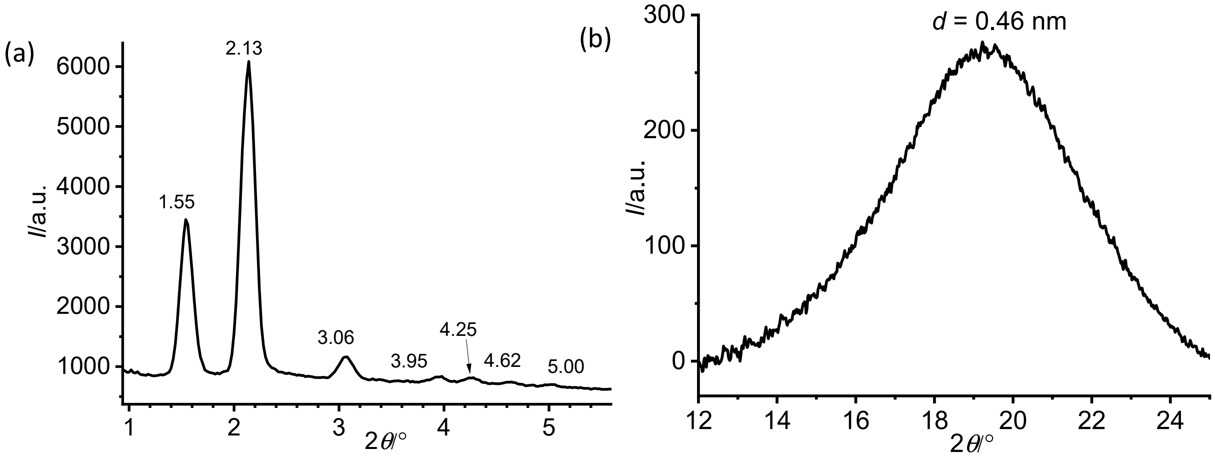

Figure S14. (a) SAXS and (b) WAXS patterns of the Col_{rec}/*c*2*mm* phase of **1/30** at *T* = 110 °C.

Table S3. Numerical XRD data of the Col_{rec}/*c*2*mm* phase of **1/30** at *T* = 110 °C.

**2*θ* /°**	***d*obs. /nm**	***d*calc. /nm**	**Δ**	***hk***
1.547	5.709	5.709	0.00	20
2.134	4.139	4.139	0.00	11
3.065	2.883	2.890	0.01	31
3.947	2.239	2.221	0.02	02
4.250	2.079	2.070	0.01	22
4.617	1.914	1.918	0.00	32
5.001	1.767	1.753	0.01	42
19.269	0.461			

Col_{rec}/*c*2*mm*: a_{rec} = 11.42 nm. b_{rec} = 4.44 nm

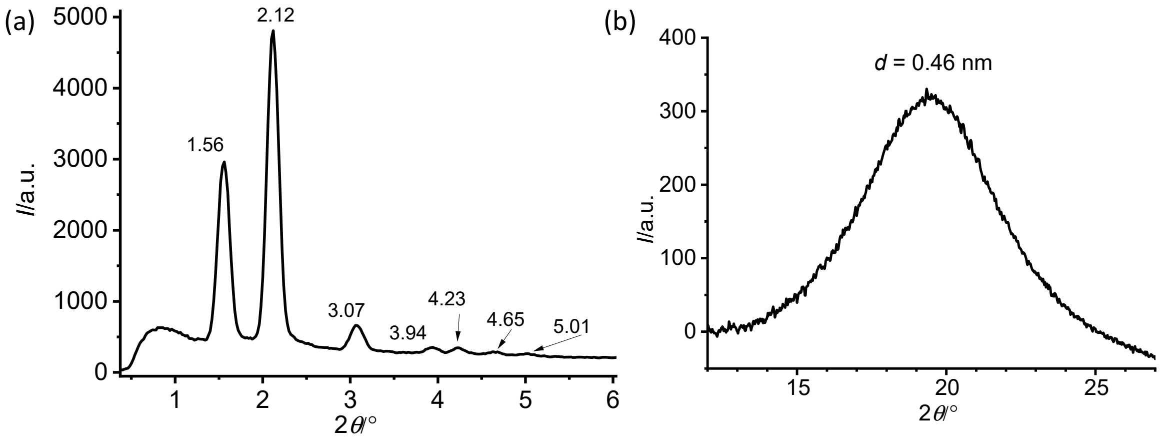

Figure S15. (a) SAXS and (b) WAXS patterns of the $Col_{rec}/c2mm$ phase of **1/32** at T = 110 °C.

Table S4. Numerical XRD data of the $Col_{rec}/c2mm$ phase of **1/32** at T = 110 °C.

**2θ /°**	$d_{obs.}$ **/nm**	$d_{calc.}$ **/nm**	**Δ**	***hk***
1.556	5.679	5.679	0.00	20
2.117	4.173	4.173	0.00	11
3.074	2.874	2.840	0.03	40
3.936	2.245	2.244	0.00	02
4.229	2.089	2.087	0.00	22
4.648	1.901	1.893	0.01	60
5.007	1.765	1.760	0.01	42
19.409	0.457			

$Col_{rec}/c2mm$: a_{rec} = 11.36 nm. b_{rec} = 4.49 nm

Table S5. Numerical XRD data of the $Col_{rec}/p2gg$ phase of **1/34** at T = 137 °C.

q	*d*-value	d_{calc}	hk	Δ	intensity	phase
0.82	7.65	7.64	11	0.01	4.3	π
0.94	6.71	6.71	20	0.00	8.7	0
1.15	5.44	5.44	21	0.00	100.0	π
1.35	4.65	4.65	12	0.00	49.9	0
1.43	4.39	4.39	12	0.00	34.8	π
1.56	4.03	4.03	31	0.00	6.4	0
1.65	3.82	3.82	22	0.00	2.4	π
1.88	3.35	3.36	40	-0.01	3.8	0
1.95	3.22	3.22	32	0.00	6.7	0
1.99	3.15	3.16	41	-0.01	8.1	π
2.08	3.02	3.02	13	0.00	7.2	π
2.24	2.81	2.81	23	0.00	1.3	0
2.32	2.71	2.72	42	-0.01	9.6	0
2.44	2.58	2.58	51	0.00	0.5	/
2.47	2.54	2.55	33	-0.01	8.4	0
2.71	2.32	2.33	04	-0.01	0.4	/
2.77	2.27	2.28	43	-0.01	5.5	π
2.82	2.23	2.24	60	-0.01	1.9	π
2.87	2.19	2.20	24	-0.01	3.2	0
3.05	2.06	2.06	34	0.00	0.1	/
3.10	2.03	2.03	53	0.00	0.5	/
3.13	2.01	2.02	62	-0.01	4.2	0
3.30	1.91	1.91	44	0.00	0.03	/
3.36	1.87	1.88	71	-0.01	0.2	/
3.42	1.84	1.84	15	0.00	2.4	0
3.47	1.81	1.81	63	0.00	2.7	π
3.57	1.76	1.76	54	0.00	1.8	/
3.82	1.64	1.63	73	0.01	1.0	/
3.87	1.62	1.63	45	-0.01	1.5	/
3.91	1.61	1.61	64	0.00	1.4	/

$Col_{rec}/p2gg$: a_{rec} = 13.42 nm, b_{rec} = 9.30 nm

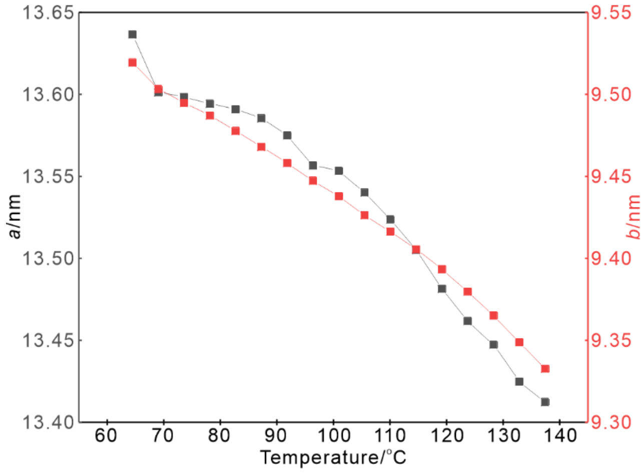

Figure S16. Temperature dependence of the **1/34** lattice parameters; as typical for honeycomb phases the increasing orientational order parameter of the *p*-terphenyl rods at reduced temperature provides an expansion of the honeycomb cells; this is associated with a small increase of n_{wall} from 2.0 at 137 °C to 2.1 at 70 °C (Table S7) to fill the larger cells.

Table S6. Numerical lattice parameters of **1/34** upon cooling.

T/°C	a/nm	b/nm	n_{wall}
137	13.41	9.33	2.04
133	13.42	9.35	2.05
128	13.45	9.37	2.05
124	13.46	9.38	2.06
119	13.48	9.39	2.06
115	13.51	9.41	2.07
110	13.52	9.42	2.08
105	13.54	9.43	2.08
101	13.55	9.44	2.08
96	13.56	9.47	2.09
92	13.57	9.46	2.09
87	13.59	9.47	2.10
83	13.59	9.48	2.10
78	13.59	9.49	2.10
74	13.60	9.49	2.10
69	13.60	9.50	2.11
64	13.64	9.52	2.12

Table S7. Calculation of the molecular volume (V_{mol}). the area of a 2D unit cell (A_{cell}). the volume of the hypothetical unit cells (V_{cell}) and number of molecules in these unit cells (n_{cell}) and in the honeycomb walls (n_{wall})[a]

Compd.	Phase	T (°C)	a,b /nm)	A_{cell} /nm^2	$V_{cell,\,cr}$/ nm^3	$V_{cell\,,LC}$ / nm^3	V_{mol} /nm^3	n_{cell}	n_{wall}
**1/24**	*p6mm*	140	4.3	16.0	7.2	6.4	1.12	5.75	1.9
**1/26**	*c2mm*	110	12.0; 4.7	56.4	25.4	22.66	1.17	19.4	2.4
**1/30**	*c2mm*	140	11.4; 4.4	50.2	22.6	20.2	1.27	15.9	2.0
**1/32**	*c2mm*	140	11.4; 4.5	51.3	23.1	20.6	1.32	15.6	2.0
**1/34**	*p2gg*	137	13.4; 9.3	127.8	56.1	50.1	1.37	36.6	2.0
**1/34**	*p2gg*	70	13.6; 9.5	129.2	58.1	51.9	1.37	37.9	2.1

[a] $V_{cell} = A_{cell} \times h$ with h = 0.45 nm, where $A_{cell} = a \times b$ for the rectangular phases and 0.866 a^2 for *p6mm*; V_{mol} = volume for a single molecule as calculated using crystal volume increments; [S5] n_{cell} = number of molecules in the unit cell, calculated according to $n_{cell} = k\ V_{cell}/V_{mol}$ with k = 0.893; [S6] n_{wall} = average number of molecules in the cross section of the honeycomb walls as calculated from n_{cell} by dividing by the number of walls per unit cell (*p6mm*: 3, *c2mm*: 8, *p2gg*: 18).

S6. Additional figures, notes and discussions

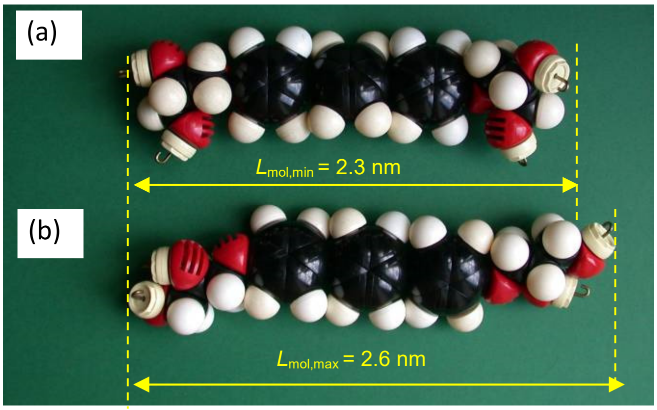

Figure S17. Molecular models showing compounds ***1/n*** (without lateral chain) in the conformations with (a) minimized and (b) maximized molecular length corresponding to $L_{mol.min}$ = 0.23 nm and $L_{mol.max}$ = 0.26 nm. respectively.

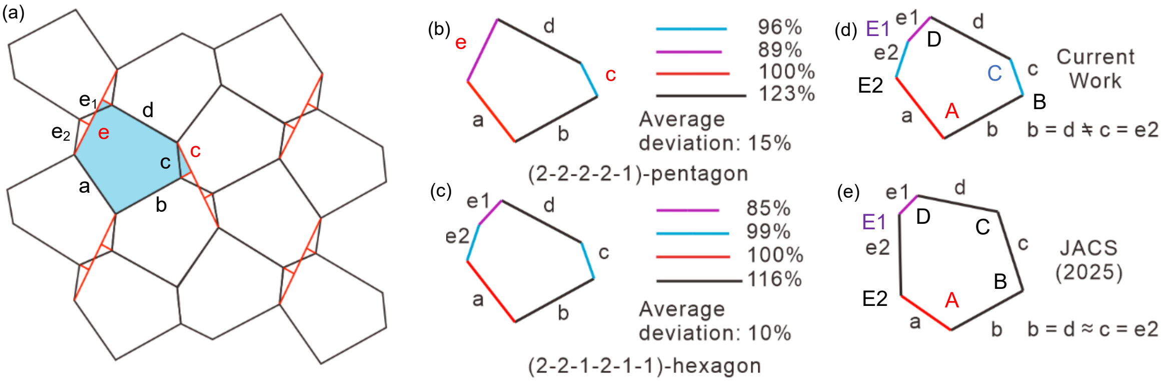

Figure S18. (a-c) Comparison of side lengths (% deviation from the molecular length of 2.27 nm) in the discussed 9-hexagon and 9-pentagon tilings of **1/34**; (d, e) shows the comparison of the edge-to-edge tilings by irregular hexagons reported (d) in this work and (e) for a COF network in ref. [S7], both forming a tiling with *p*2*gg* plane group. There are only 3 possible types of irregular hexagons which can tile the Euclidean plane, requiring specific conditions for side lengths and vertex angles. [S8, S9] In (d) and (e) the sides **e2** = **c**, **b** = **d** and the inner angles at the vertices are **E2** + **A** + **C** = 360°, both fulfilling the conditions of a *type-2 hexagon* tiling. [S8,S9] We note that in ref. [S7] the irregular hexagonal tiling with (e) was designated as *type-III,* which might lead to confusion. In *type-3 hexagons* - as defined in refs.[S8,S9a,b] - every second vertex is 120° and there are three pairs of adjacent sides with identical length, i.e. the conditions for the edges would, for example, be **e2** = **a**, **b** = **c** and **d** = **e1** with each pair enclosing one of the 120° vertices (**E2** = **B** = **D** = 120°). Such tiles would lead to a tiling pattern with *p*3 or *p*31*m* plane group. This is very different from the hexagons (e) reported in [S7]. (The *type-1* hexagon would have the conditions **e2** = **c** and **E2** + **A** + **B** = 360°, note that the numbering of the vertices and edges is here according to the sequence of long and short chains (2-2-1-2-1-1) and different from that used in refs. [S8,S9]) The tile (e) could also be regarded as a non-regular pentagon where the short edge (**e1**) is considered as an expanded vertex fusing two 3-way junctions to one slightly expanded 4-way junction, leading to a tessilation related to the edge-to-edge Cairo-type pentagon tiling. [S10] In contrast, the hypothetical pentagon tiling of the (2-2-2-2-1)-pentagons in (b) would not be edge-to-edge (see (a) and Fig. 6c).

S7. References

S1 A. Lehmann, A. Scholte, M. Prehm, F. Liu, X. Zeng, G Ungar and C. Tschierske, Soft Rectangular Sub-5 nm Tiling Patterns by Liquid Crystalline Self-Assembly of T-Shaped Bolapolyphiles, *Adv. Funct. Mater*. **2018**, 1804162.

S2 A. Scholte, S. Hauche, M. Wagner, M. Prehm, S. Poppe, C Chen, F. Liu, X. Zeng, G Ungar and C. Tschierske, A self-assembled liquid crystal honeycomb of highly stretched (3-1-1)-hexagons, *Chem. Commun.* **56** (2020) 62-65.

S3 R. Dunkel, M. Hahn, K. Borisch, B. Neumann, H.-H. Rüttinger, C. Tschierske, Determination of the water content of amphiphilic liquid crystals by coulometric Karl Fischer titration, *Liq. Cryst.* **24** (**1998**) 211-213.

S4 D. Joachimi, G. Lattermann, M. Schelhorn, C. Tschierske, P. Zugemnmaier, Investigation of the crystal structure of two amphiphilic diols, *Liq. Cryst*. **18** (1995**)** 303-307.

S5 A. Immirizi and B. Perini, *Acta Cryst.* **A33** (1977) 216-218.

S6 A. I. Kitaigorodski, *Molekülkristalle*, Akademieverlag: Berlin, Germany, **1979**.

S7 H. Li, X-R. Ren, Y. Wang, D. Zhang, Z. Wang, L-T. Wei, X-L Wang, D. Wang, Reticular Design and Synthesis of Covalent Organic Frameworks with Irregular Hexagonal Tiling, *J. Am. Chem. Soc*. **147** (2025) 10840-11545.

S8 K. Reinhardt, Über die Zerlegung der Ebene in Polygone, Ph.D. Thesis, Universität Frankfurt, **1928**.

S9 a) R. B. Kershner, On paving the plane, *Am. Math. Monthly*, 1968, 75. 839-844; b) C. Zong, Can You Pave the Plane with Identical Tiles? *Notices Am. Math. Soc.* **67** (2020) 635-646.; c) Double Donut, Irregular Hexagon Tessellation, Vacuolation, www.youtube.com/watch?v=kRzSif605hc; accessed: 28.07.2025

S10 Y. Liu, L. Yuan, W. Chi, W.-K. Han, J., Zhang, H. Pang, Z, Wang, Z.-G. Du, Cairo pentagon tessellated covalent organic frameworks with mcm topology for near-infrared phototherapy, *Nat. Commun*. **15** (2024) 7150.
